# Efficient analysis of adverse drug events and toxicological mechanisms of newly marketed drugs by integrating pharmacovigilance and network toxicology: selumetinib as an example

**DOI:** 10.3389/fphar.2024.1432759

**Published:** 2024-08-13

**Authors:** Rui Xiong, Jing Lei, Lu Wang, Shipeng Zhang, Hengxu Liu, Hongping Wang, Tao Liu, Xiaodan Lai

**Affiliations:** ^1^ Department of Pharmacy, Jiangbei Campus of The First Affiliated Hospital of Army Medical University (The 958th Hospital of Chinese People’s Liberation Army), Chongqing, China; ^2^ Department of Pharmacy, Daping Hospital, Army Medical University, Chongqing, China; ^3^ Department of Pharmacy, The First Affiliated Hospital of Army Medical University, Chongqing, China; ^4^ Department of Infectious Diseases, Navy No.971 Hospital, Qingdao, Shandong, China

**Keywords:** food and drug administration adverse event reporting system, pharmacovigilance, network toxicology, selumetinib, neurofibromatosis

## Abstract

**Objective:**

To integrate pharmacovigilance and network toxicology methods to explore the potential adverse drug events (ADEs) and toxic mechanisms of selumetinib, and to provide a reference for quickly understanding the safety and toxicological mechanisms of newly marketed drugs.

**Methods:**

Taking selumetinib as an example, this study integrated pharmacovigilance methods based on real-world data and network toxicology methods to analyze its ADE and its potential toxicological mechanism. First, the ADE reports of selumetinib were extracted from the US Food and Drug Administration (FDA) adverse event reporting system (FAERS), and the ADE signals were detected by reporting odds ratio (ROR) and UK medicines and healthcare products regulatory agency (MHRA) methods. The ADE signals were classified and described according to the preferred terms (PTs) and system organ class (SOC) derived from the Medical Dictionary for Regulatory Activities (MedDRA). The network toxicology method was used to analyze the toxicological mechanism of the interested SOCs. The specific steps included predicting the potential targets of selumetinib using TOXRIC, STITCH, ChEMBL, CTD, SwissTargetPreditcion, and Super-PRED databases, collecting the targets of SOC using GeneCards database, conducting protein-protein interaction (PPI) analysis through STRING database, conducting gene ontology (GO) and Kyoto encyclopedia of genes and genomes (KEGG) analysis through DAVID database, and testing the molecular affinity using AutoDock software.

**Results:**

A total of 1388 ADE reports related to selumetinib were extracted, and 53 positive signals were detected by ROR and MHRA methods, of which 20 signals were not mentioned in the package insert, including ingrowing nail, hyperphosphatemia, cardiac valve disease, hematuria, neutropenia, etc. Analysis of the toxicological mechanism of six SOCs involved in positive ADE signals revealed that the key targets included EGFR, STAT3, AKT1, IL6, BCL2, etc., and the key pathways included PI3K/Akt pathway, apoptosis, ErbB signaling pathway, and EGFR tyrosine kinase inhibitor resistance, etc. Molecular docking assays showed spontaneous binding of selumetinib to key targets in these pathways.

**Conclusion:**

The pharmacovigilance analysis identified some new potential ADEs of selumetinib, and the network toxicology analysis showed that the toxic effects of selumetinib may be related to PI3K/Akt pathway, apoptosis, ErbB signaling pathway, EGFR tyrosine kinase inhibitor resistance and other pathways.

## 1 Introduction

The rapid development of world science and technology has brought great convenience to the process of drug research and development, and new drugs are constantly approved for market. Although toxicological studies and clinical safety evaluation have been conducted before the drug is marketed, some rare or delayed adverse drug events (ADEs) are difficult to discern due to the limitations of sample size and duration of medication. Post-market safety evaluation of drugs based on real-world data can help fill this gap, and the US Food and Drug Administration (FDA) adverse event reporting system (FAERS) provides an excellent choice to explore the potential ADEs (Sakaeda et al., 2013).

On the basis of comprehensive and accurate understanding of ADEs, the exploration of toxicological mechanism of specific ADEs will be conducive to avoiding and accurately dealing with consequences associated with ADEs. Due to ethical constraints, toxicological studies of drugs are usually conducted through *in vitro* and animal experiments, which are quite different from the actual toxic effects of drugs in humans. Although many ADEs can be clinically observed and proven to be associated with drug use, the toxicological mechanisms of ADEs are unclear. Until now, the methods that can be used to study the toxicological mechanisms of drugs are still very limited. In recent years, the network toxicology method derived from network pharmacology provides a feasible way to quickly and efficiently understand the toxicological mechanism of drugs (Huang, 2023; Zhao et al., 2023). Integrating pharmacovigilance and network toxicology methods to explore the ADEs and their potential toxicological mechanisms of newly marketed drugs is helpful to quickly discover the potential ADEs and their toxicological mechanisms. In this study, we will use selumetinib as an example to illustrate the executive steps of integrating pharmacovigilance and network toxicology, and to initially elucidate the potential ADE signal of selumetinib and its toxicological mechanism.

Selumetinib, a MAPK kinase (MEK) inhibitor, was approved by the FDA in April 2020 for the treatment of children with neurofibromatosis type 1 (NF1)-related symptomatic plexiform neurofibroma (Gross et al., 2020). As the first approved worldwide drug for the treatment of NF1, selumetinib has brought benefits to patients with NF1, but its safety issues in skin, eye, heart and other aspects have also caused concern (Ho et al., 2022; Gross et al., 2023). Selumetinib has been on the market for a short time and has not been widely used in many countries and regions. Therefore, the clinical application experience of selumetinib is insufficient, and doctors and patients have limited understanding of its safety. In this study, we aimed to explore the potential ADE signals of selumetinib using FAERS to provide a reference for people to understand its safety more comprehensively. In addition, the potential toxicological mechanisms of ADE associated with selumetinib also explored using network toxicology method. This study provides a new paradigm for the rapid and efficient exploration of ADEs and their potential toxicological mechanisms, contributing to a comprehensive understanding of drug safety.

## 2 Materials and methods

### 2.1 ADE data source

The ADE data of selumetinib in this study were derived from FAERS database, which serve as a publicly available database that contains spontaneous ADE reports submitted to FDA by consumers, healthcare professionals, and manufacturers. Data from FAERS can be extracted via the OpenVigil2.1 platform (http://h2876314.stratoserver.net:8080/OV2/search), an open pharmacovigilance tool, which provides filtering criteria on the input side and output side, respectively (Böhm et al., 2012). The filtering criteria on the input side include drug name (primary suspect, secondary suspect, concomitant, and interacting), adverse event (supporting search at different levels), time of report submission, gender and age of the patient, indication, country or region, and outcome of the report. Filters on the output include raw data, frequency and frequentist methods, which means that raw data and data after disproportionality analysis can be extracted. Disproportionality analysis was performed based on the reporting odds ratio (ROR) and proportional reporting ratio (PRR).

### 2.2 Data processing

Using “selumetinib” as the search keyword, we extracted all ADE reports associated with selumetinib from 10 April 2020 to 31 March 2024 through the Openvigil2.1 platform. The filtering criteria on the input side such as gender, age, reporting country and reporting year were set respectively and the frequentist methods was used as the output format to extract ADE reports of selumetinib for descriptive analysis of the overall situation of ADE reports. The extracted reports were screened individually by two researchers independently to remove entries that were not related to the ADE, i.e., not caused by medication use, such as product-related issues and various medical practices. If the reports deleted by the two researchers were inconsistent, a third researcher was invited to participate in the discussion together and a final consensus was reached. To mitigate the confounding effects of “indication bias” (i.e., the indication of the drug is reported as an ADE), PTs associated with neurofibromatosis was removed from the analysis. After invalid reports were excluded, ADEs were classified and standardized according to the preferred terms (PTs) and system organ class (SOC) derived from the Medical Dictionary for Regulatory Activities (MedDRA) terminology.

### 2.3 Disproportionality analysis

This study used the disproportionation analysis commonly used in pharmacovigilance studies, which compares the proportion of adverse reactions occurring between a particular drug and all other drugs, to identify signals of drug-related adverse events. In this study, the reporting odds ratio (ROR) and UK medicines and healthcare products regulatory agency (MHRA) methods were used to detect ADE signals associated with selumetinib. The values of ROR and PRR reflect the strength of the association between ADE signals and drugs, and the positive ADE signals were screened according to previous described, and a signal was determined to be positive only when it met the criteria of both methods (Xiong et al., 2023).

### 2.4 Network toxicological analysis

Network toxicology is a method for rapid prediction of drug toxicity mechanism based on system biology theory and network pharmacology technology. In this study, the predicted targets of selumetinib were obtained from TOXRIC, STITCH, ChEMBL, Comparative Toxicogenomics Database (CTD), SwissTargetPreditcion, and Super-PRED databases, and the relevant targets of key SOCs were collected through GeneCards (Stelzer et al., 2016; Szklarczyk et al., 2016; Daina et al., 2019; Gallo et al., 2022; Davis et al., 2023; Wu et al., 2023; Zdrazil et al., 2024). Venn diagram was used to identify the intersection of selumetinib prediction targets and SOC-associated targets, which serve as potential toxicity targets of selumetinib associated SOCs. A protein-protein interaction (PPI) network map of potential toxicity targets was constructed using the STRING database and Cytoscape3.7.2 software to identify key targets (Szklarczyk et al., 2023). Gene ontology (GO) and Kyoto encyclopedia of genes and genomes (KEGG) enrichment analysis of potential toxicity targets for key SOCs were performed using the DAVID database and visualized via the bioinformatics (https://www.bioinformatics.com.cn/) and omicshare platforms (https://www.omicshare.com/) (Sherman et al., 2022).

### 2.5 Molecular docking

Virtual molecular docking with reference to previous methods reveals the affinity of drugs to key target proteins (Xiong et al., 2020). Briefly, the molecular structure of the drug and the target protein is downloaded from the PubChem database and the RCSB PDB database (https://www.rcsb.org) respectively. Chem3D software was used to minimize the energy of the ligand compounds and convert them into mol2 format files. Water molecules, metal ions, and ligands bound to protein macromolecules were removed and then converted to PDB files. Then the AutoDock software was employed to perform semi-flexible (i.e., protein macromolecules are maintained as rigid structures and small molecular ligands as flexible structures) molecular docking according to Lamarckian genetic algorithm. Binding energy was used to evaluate the affinity between ligand (drug) and receptor (target protein).

### 2.6 Statistical analysis

The ROR and PRR values were calculated according to formulas by referring to the previous methods using Microsoft Excel 2019 software. If the number of ADE reports ≥3, the lower bound of the 95% confidence interval (CI) of the ROR value exceeds 1, the PRR ≥2, and the χ^2^ ≥ 4, it is judged as a positive ADE signal (Xiong et al., 2023). The *P* values in GO and KEGG enrichment analysis were obtained using the DAVID database, and the topological parameters in network analysis were calculated using the CytoNCA plug-in in cytoscape3.7.2 software. *P* < 0.05 was considered statistically significant.

## 3 Results

### 3.1 Descriptive analysis of selumetinib ADE reports

**FIGURE 1:**
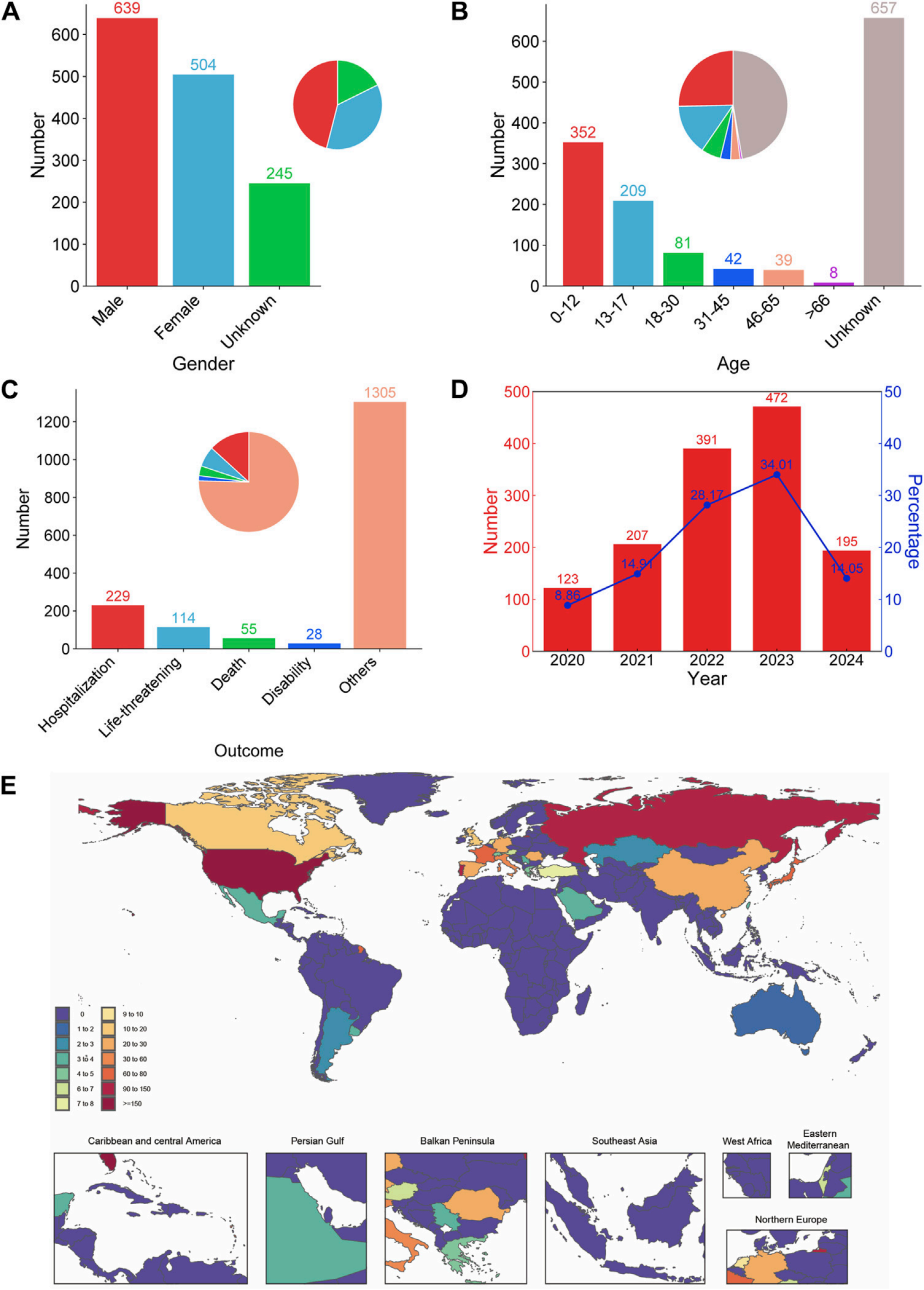
Demographic characteristics of ADE reports for selumetinib. Distribution of gender **(A)**, age **(B)**, reported outcome **(C)**, year **(D)**, and country/region **(E)** of selumetinib ADE reports.

A total of 4,678,471 reports were extracted from FAERS using OpenVigil2.1 between 10 April 2020 and 31 March 2024, of which 1,388 were related to selumetinib. Among the reports that recorded gender, males accounted for a higher proportion than females (Figure 1A). There were 657 (47.33%) of the reports did not record age. Among the reports with known age, 0–12 years old accounted for the largest proportion, followed by 13–17 years old. The cumulative proportion of reports of patients under 18 years old was more than 40%, and about 12.25% of the reports of patients over 18 years old (Figure 1B). In the reports of serious outcomes, hospitalization (initial or prolonged) accounted for the largest proportion (229, 16.50%), followed by life-threatening (114, 8.21%), death (55, 3.96%), disability (28, 2.02%), and most of the reported outcomes were not serious (Figure 1C). The number of ADE reports submitted to FAERS has consistently increased year by year since the approval of selumetinib for marketing, with the highest number of reports being submitted in 2023 (472, 34.01%). 195 reports were submitted in the first quarter of 2024, and with this trend, the number of reports for all of 2024 will likely exceed that of 2023 (Figure 1D). In the country/region distribution, most reports from the United States, accounted for 59.73% (829 of 1,388), followed by Russian Federation (121, 8.72%), Portugal (91, 6.56%), France (72, 5.19%), and Japan (67, 4.83%) while other countries report less, accounting for less than 15% (Figure 1E).

### 3.2 ADE signal of selumetinib at PT level

**FIGURE 2:**
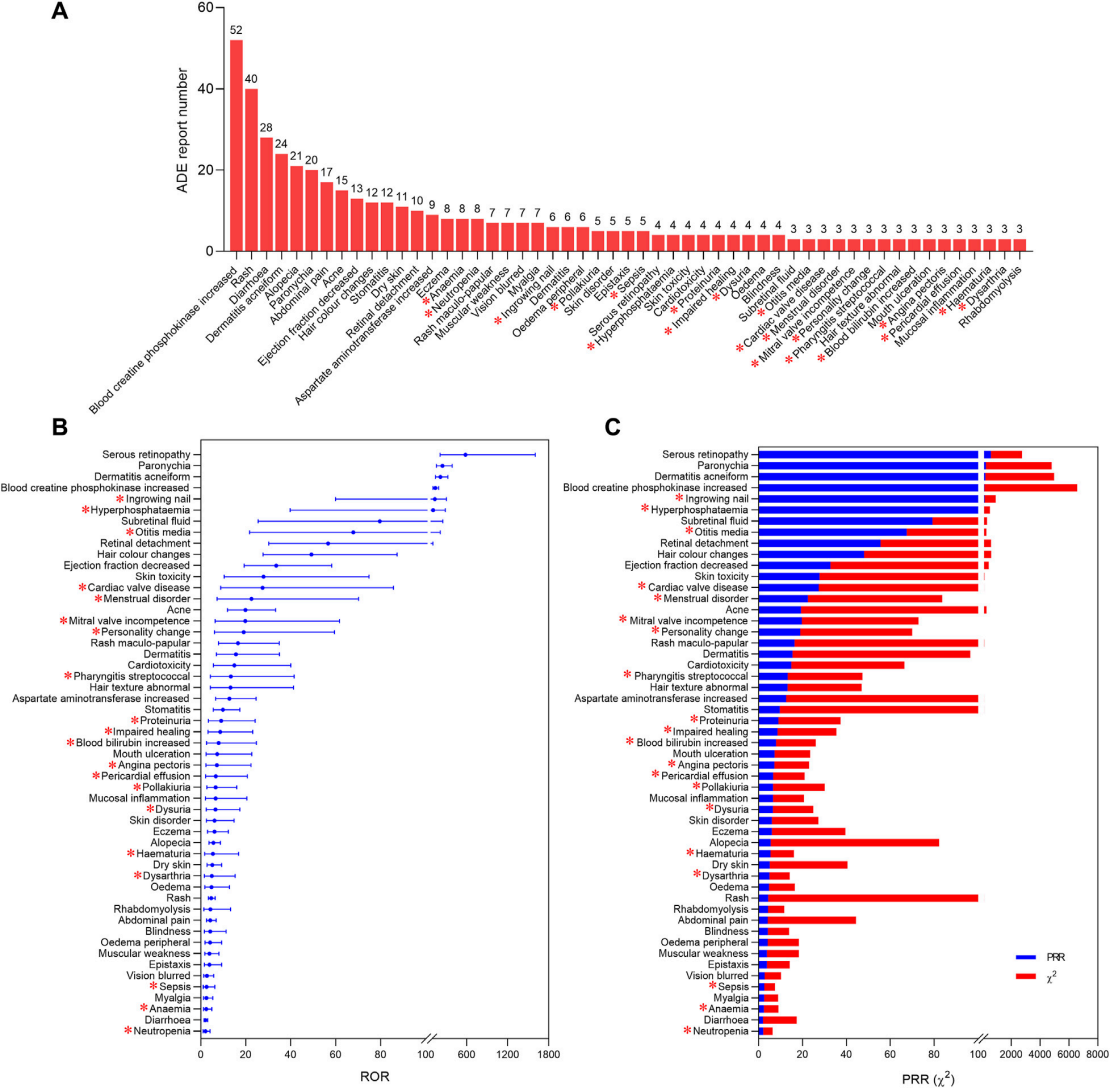
Positive ADE signals of selumetinib at PT level. The report number **(A)**, ROR values **(B)**, PRR values and chi-square values **(C)** of 53 positive ADE signals. * indicates ADE signals not mentioned by the package insert.

After screening, 1,187 reports were retained (Supplementary Table S1) and 201 were deleted (Supplementary Table S2). According to the detection criteria of ROR and MHRA methods, 53 positive ADE signals were identified (Figure 2). The five ADE signals with the highest number of reports were blood creatine phosphokinase increased (52, 4.38%), rash (40, 3.37%), diarrhoea (28, 2.36%), dermatitis acneiform (24, 2.02%), and alopecia (21, 1.77%) (Figure 2A), while the five ADE signals with the highest signal strength were serous retinopathy, paronychia, dermatitis acneiform, blood creatine phosphokinase increased, and ingrowing nail. Among the 53 positive signals, the ADEs mentioned in the package insert such as cardiac disorders, eye disorders, diarrhea, rash, and rhabdomyolysis were accurately identified, but some positive signals were not mentioned in the package insert, including ingrowing nail, hyperphosphataemia, otitis media, cardiac valve disease, menstrual disorder, mitral valve incompetence, personality change, pharyngitis streptococcal, proteinuria, impaired healing, blood bilirubin increased, angina pectoris, pericardial effusion, pollakiuria, dysuria, haematuria, dysarthria, sepsis, anaemia, neutropenia.

### 3.3 SOC associated with positive ADE signals

**FIGURE 3:**
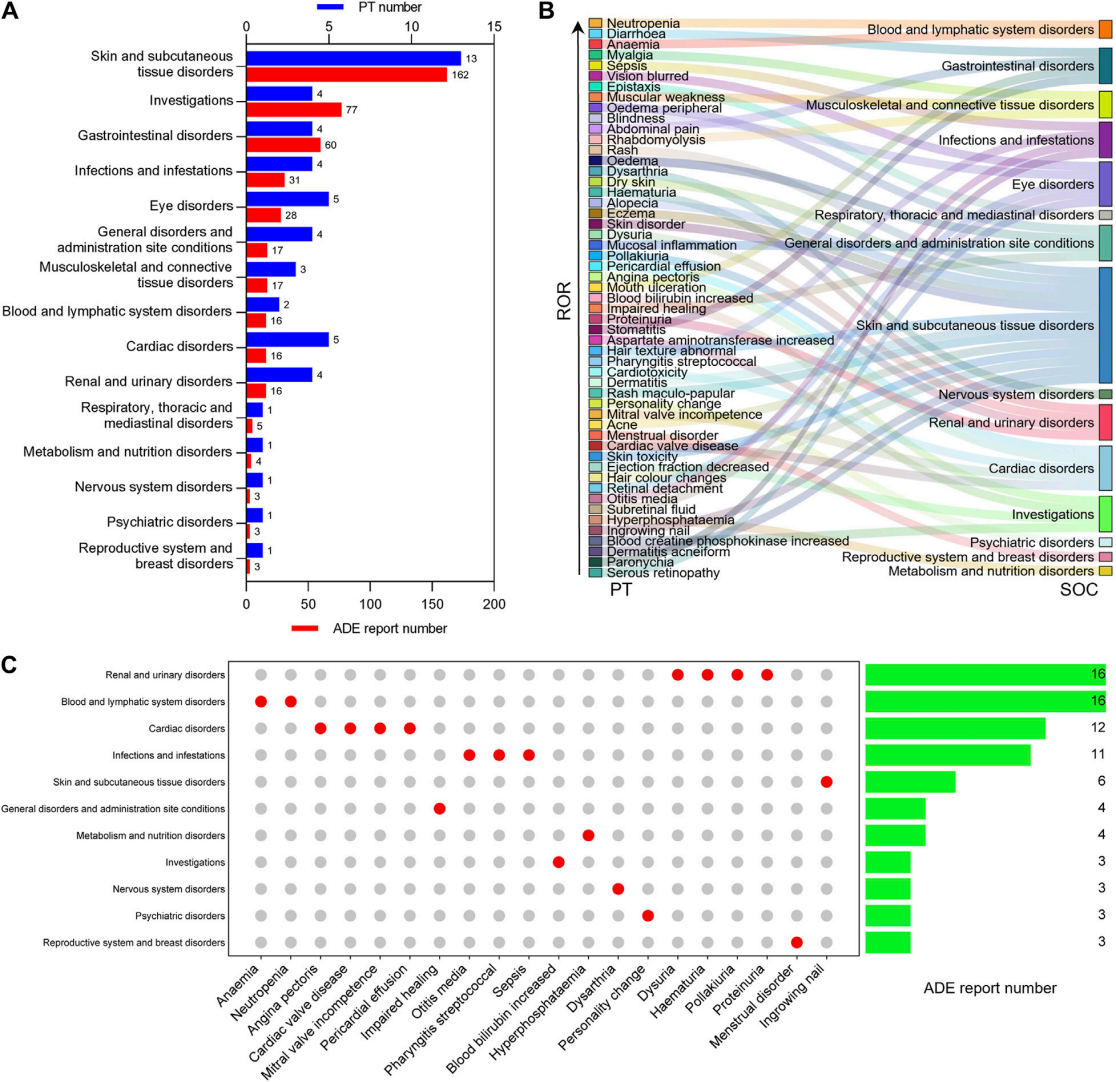
Distribution of positive ADE signals at SOC level. **(A)** The number of PT and ADE reports contained in the SOC associated with positive ADE signal of selumetinib. **(B)** Sankey diagram of the relationship between positive ADE signal and SOC. **(C)** Number of reports contained in SOCs involved in new potential ADE signals.

To understand the distribution of selumetinib ADEs at the SOC level, we mapped the 53 identified positive ADE signals to SOCs based on the medical terminology set in MedDRA, and found that 15 SOCs were involved, with most PT numbers and ADE reports being skin and subcutaneous tissue disorders (13, 162), followed by investigations (4, 77), gastrointestinal disorders (4, 60), infections and infestations (4, 31), and eye disorders (5, 28) (Figures 3A,B). Among these SOCs involved, 10 were related to ADE recorded or mentioned in the package insert, and five were not mentioned, namely, metabolism and nutrition disorders, renal and urinary disorders, blood and lymphatic system disorders, reproductive system and breast disorders, and psychiatric disorders. The 20 new potential ADE signals involved 11 SOCs (Figure 3C), six of which were already mentioned in the package insert. Among the 11 SOCs involved in new potential ADE signals, renal and urinary disorders and blood and lymphatic system disorders accounted for the largest proportion of reports, followed by cardiac disorders, infections and infestations, and skin and subcutaneous tissue disorders (Figure 3C).

### 3.4 PPI network analysis of toxicity targets of selumetinib key SOCs

**FIGURE 4:**
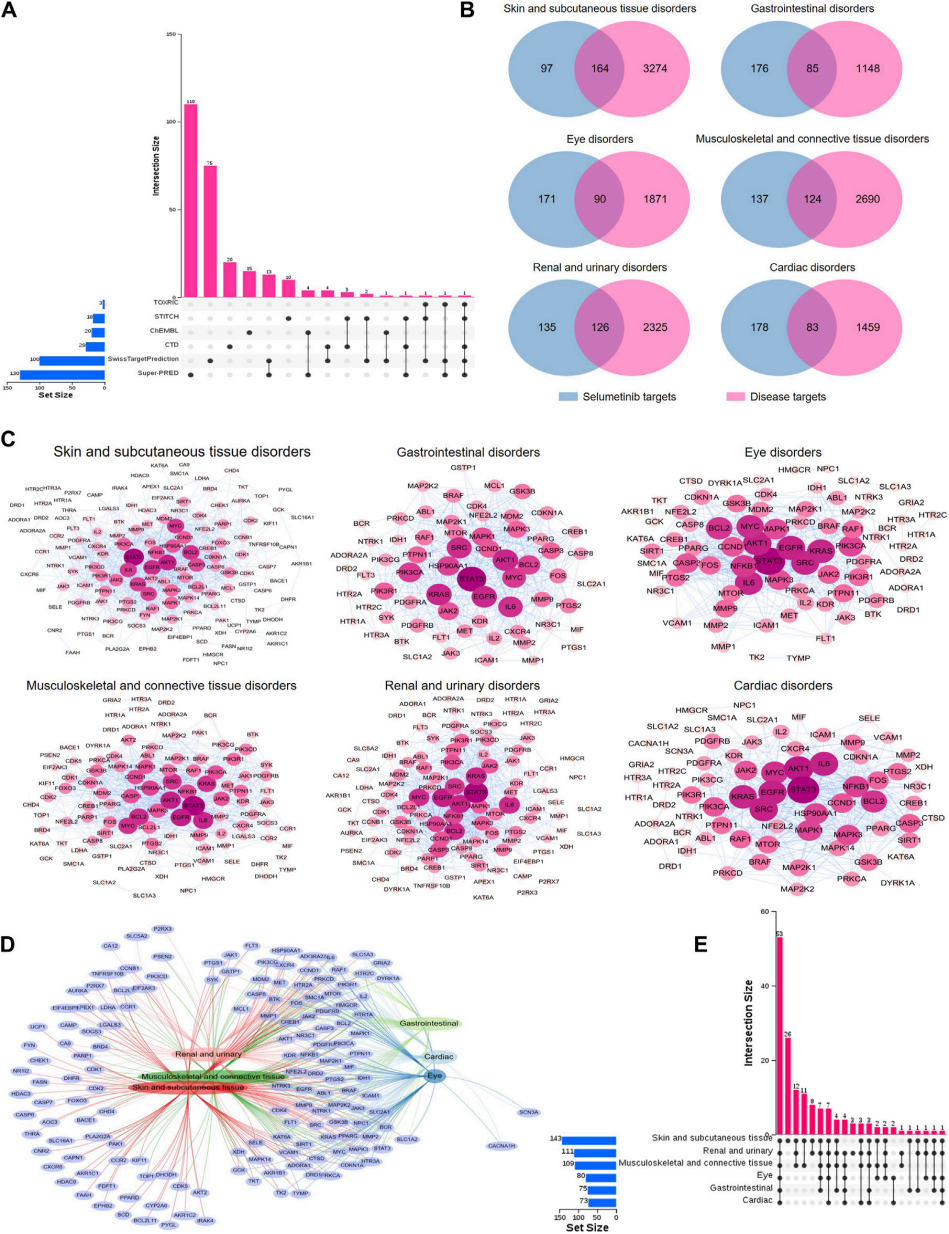
PPI networks of potential toxic targets of selumetinib. **(A)** Sources of predicted targets of selumetinib. **(B)** Venn diagram of prediction targets and key SOCs of selumetinib. **(C)** PPI network map of common targets of selumetinib and six key SOCs. **(D)** Network Venn diagram of potential toxicity targets of six key SOCs. **(E)** Upset diagram of potential toxicity targets of six key SOCs.

In order to explore the toxicological mechanism of the occurrence of ADE associated with selumetinib, the SOCs with PT number greater than three and ADE reports greater than 10 were selected as key SOCs and the network toxicology was employed to analyze the toxicological mechanism. Since the investigations, infections and infestations, and general disorders and administration site conditions are not for a specific systemic organ disease, toxicological mechanism analysis was not considered. We collected a total of 261 prediction targets of selumetinib from six databases, some of which could be predicted by multiple databases (Figure 4A). Associated targets of six key SOCs were collected from GeneCards database and common targets with selumetinib were identified through Venn maps, which may be potential toxic targets for the occurrence of selumetinib ADEs (Figure 4B). The PPI network was further constructed through STRING database and Cytoscape software, and the topological analysis showed that the six SOCs had similar key nodes, including EGFR, STAT3, AKT1, IL6, BCL2 and so on (Figure 4C). The network Venn diagram and the upset diagram showed that there were many common potential toxicity targets for the 6 SOCs, for example, the 6 SOCs shared 53 targets, skin and subcutaneous tissue disorders, renal and urinary disorders, and musculoskeletal and connective tissue disorders shared 12 targets (Figures 4D,E). There are also some targets that were unique to certain SOCs, such as 26 targets associated only with skin and subcutaneous tissue disorders and 3 targets associated only with renal and urinary disorders.

### 3.5 GO and KEGG enrichment analysis of potential toxic targets of key SOCs of selumetinib

**FIGURE 5:**
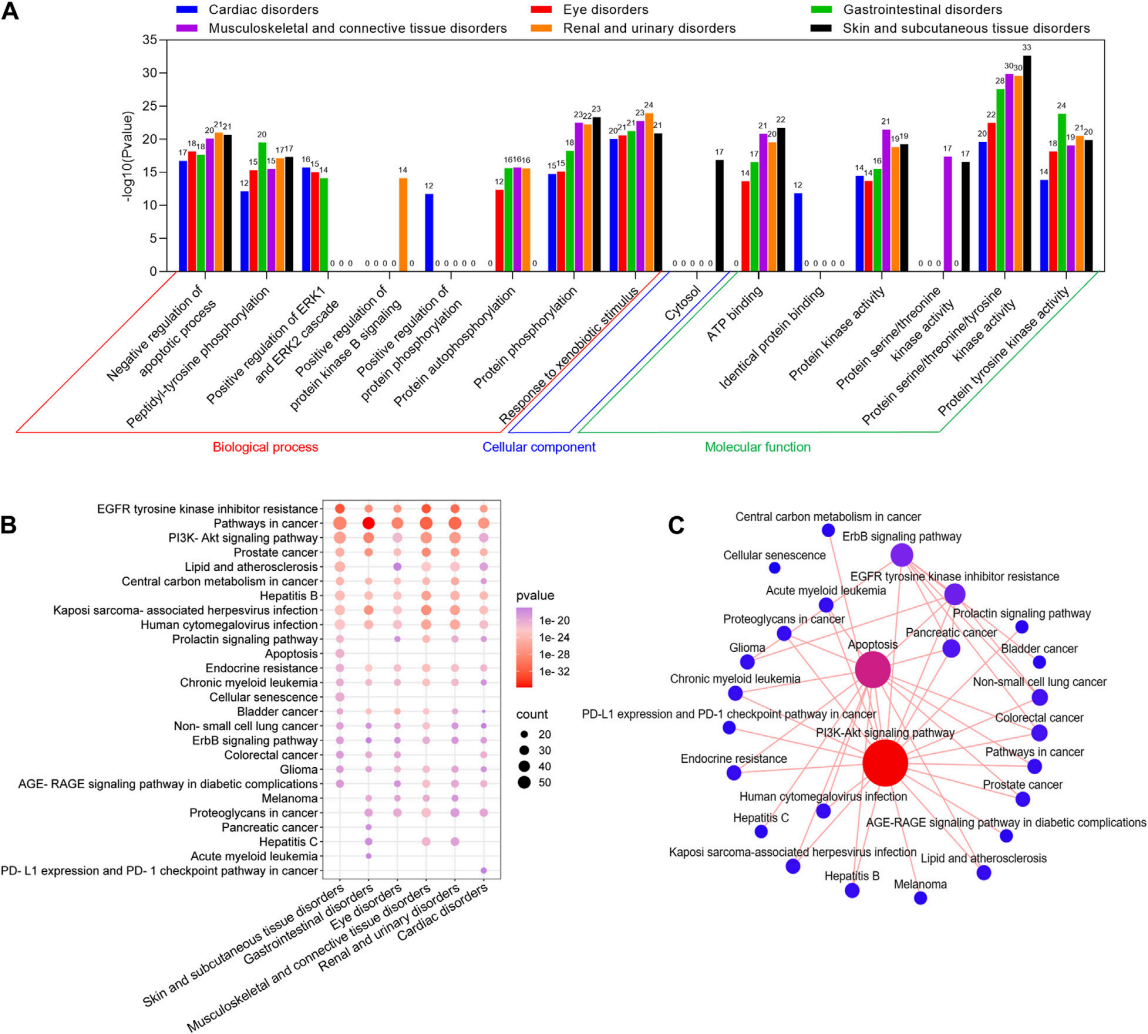
GO and KEGG enrichment analysis of potential toxic targets of key SOCs of selumetinib. GO functional **(A)** and KEGG pathway **(B)** enrichment analysis of potential toxic targets of six key SOCs. **(C)** Interaction network of the top 20 significant pathways in KEGG enrichment analysis of the toxicity targets of six key SOCs.

To further explore the role of toxic targets of the key SOCs of selumetinib in regulating cellular life processes, the GO and KEGG enrichment analysis was performed through DAVID database. In the GO enrichment analysis of the toxic targets of the 6 key SOCs, the top 10 GO items included 8 biological processes (negative regulation of apoptotic process, peptidyl-tyrosine phosphorylation, positive regulation of ERK1 and ERK2 cascade, positive regulation of protein kinase B signaling, positive regulation of protein phosphorylation, protein autophosphorylation, protein phosphorylation, response to xenobiotic stimulus), 1 cell component (cytosol), and 6 molecular functions (ATP binding, identical protein binding, protein kinase activity, protein serine/threonine kinase activity, protein serine/threonine/tyrosine kinase activity, protein tyrosine kinase activity) (Figure 5A). In KEGG enrichment analysis of toxic targets, the top 20 significant pathways of six key SOCs were similar, mainly including EGFR tyrosine kinase inhibitor resistance, pathway in cancer, PI3K-Akt signaling pathway, etc (Figure 5B). Since there is usually cross-regulation between KEGG pathways, we plotted the KEGG pathway network between the top 20 significant pathways of 6 key SOC. The results clearly showed that PI3K-Akt pathway had the highest degree value, followed by apoptosis, ErbB signaling pathway, and EGFR tyrosine kinase inhibitor resistance, indicating that these pathways were the common key pathways in these SOCs (Figure 5C).

### 3.6 SOC-pathway-target network construction and key toxicity target identification

**FIGURE 6:**
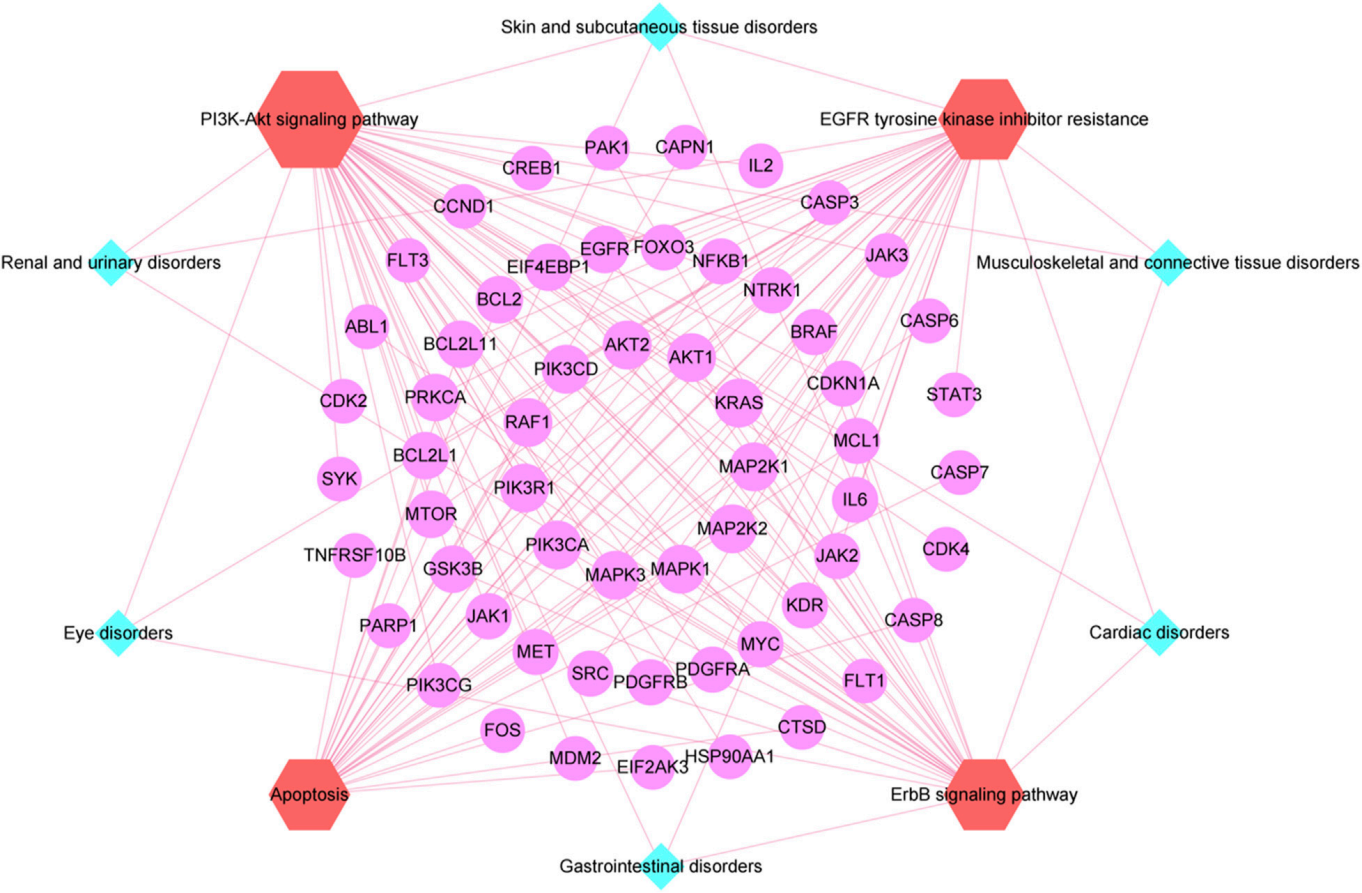
SOC-pathway-target network. The indigo diamonds represent the six key SOCs related to selumetinib, the red hexagons represent the four important pathways related to the six key SOCs related to selumetinib, and the purple circles represent the targets involved in the four important pathways.

To precisely identify the core targets in 4 key KEGG pathways associated with 6 key SOCs, the SOC-pathway-target network was constructed using Cytoscape software (Figure 6). Topological analysis of SOC-pathway-target network revealed 11 targets with Degree value = 4, namely, AKT1, KRAS, MAP2K1, MAP2K2, MAPK1, MAPK3, PIK3CA, PIK3R1, RAF1, PIK3CD, AKT2, indicating that these targets were associated with all 4 key KEGG pathways, which demonstrated that these targets may be key toxicity targets for selumetinib.

### 3.7 Molecular docking of selumetinib to key toxicity targets

**FIGURE 7:**
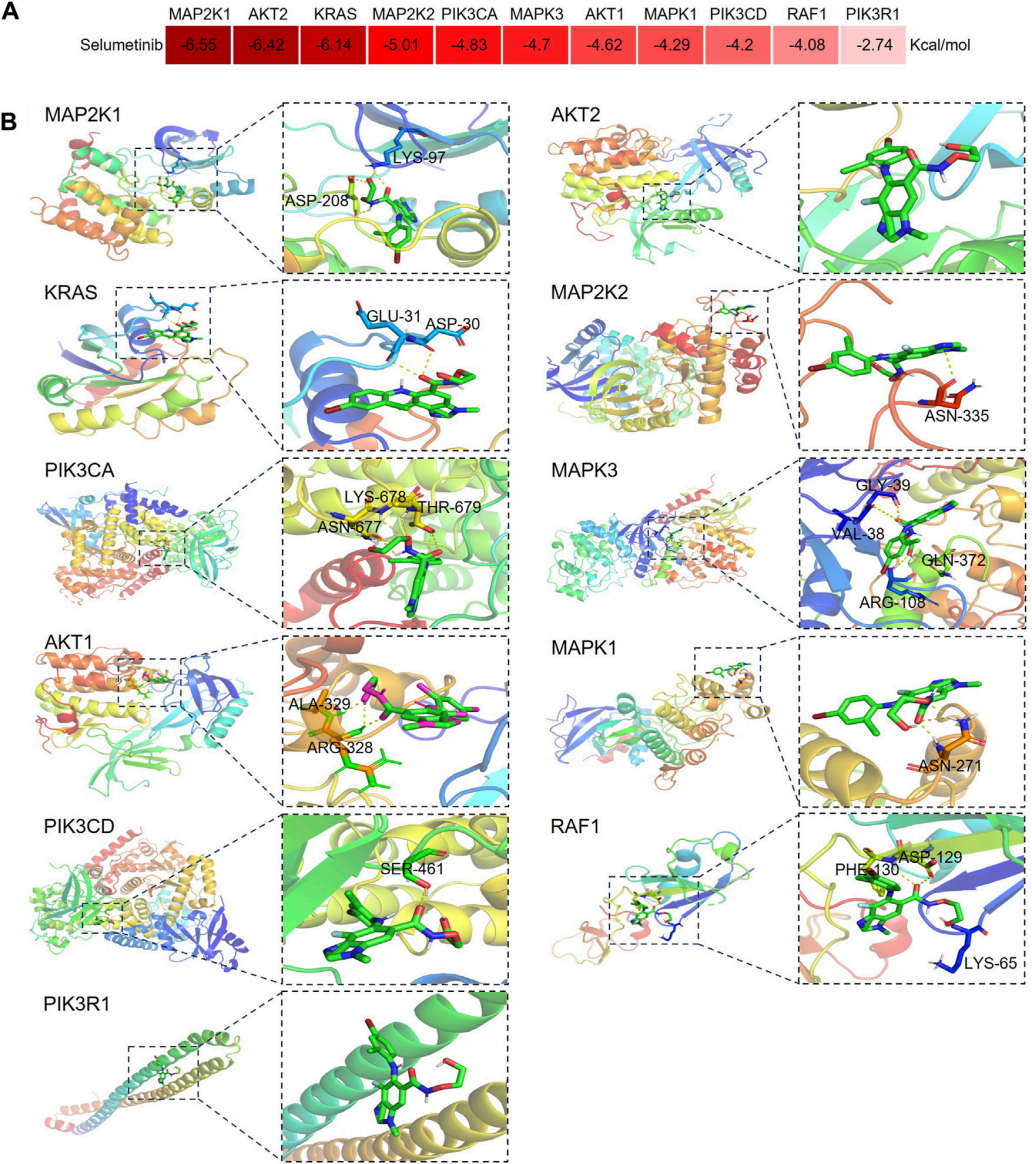
Diagram of molecular docking patterns between selumetinib and key toxicity targets. **(A)** Heat map of binding energy between selumetinib and proteins expressed by key toxicity genes. **(B)** Binding site of selumetinib to proteins expressed by key toxicity genes.

To further investigate the regulatory effects of selumetinib on these key toxicity targets, the affinity of selumetinib with proteins expressed by key toxicity targets was explored by molecular docking technology. The results showed that the binding energies of selumetinib to these key toxicity target proteins were all lower than 0 kcal/mol, suggesting that selumetinib binds spontaneously to these target proteins (Figure 7A). Selumetinib binds to AKT2 and PI3KR1 with van der Waals forces and forms hydrogen bonds with other proteins. Figure 7B shows the site of selumetinib binding to the protein and identifies the amino acid residues that form hydrogen bonds with selumetinib.

## 4 Discussion

### 4.1 Analysis of pharmacovigilance signals

Neurofibromatosis is a rare genetic and tumor-prone disorder affecting the nervous system, including NF1, NF2, and schwannomatosis (Anderson et al., 2021). NF1 is the most typical neurofibromatosis, accounting for about 90% of all cases and occurs in about 1/3,000, and characterized by multiple café au lait macules (CALMs), skinfold freckling (also known as the lentiginous macules), iris Lisch nodules, tumors of the nervous system, and other features caused by mutations in the NF1 gene on chromosome 17 (Legius et al., 2021). Some features of NF1 can be present at birth, but most manifestations emerge with age. Current treatment options for NF1 are limited, and surgical treatment and symptomatic treatment aimed at alleviating symptoms, reducing tumor recurrence and complications, and improving quality of life are the main approaches to deal with NF1 (Saleh et al., 2023).

Neurofibromin, the product of the NF1 gene, has previously been found to function primarily as a GTPase-activating protein (GAP) that negatively regulates the RAS/MAPK pathway activity by accelerating the hydrolysis of RAS-bound GTP (Gutmann et al., 2017). The loss of function of NF1 gene leads to the upregulation of the activity of RAS/MAPK pathway, which leads to the uncontrolled cell proliferation and growth (Anderson et al., 2021). Therefore, inhibiting RAS/MAPK pathway becomes an important means to compensate for the loss of function of NF1 gene. Based on this strategy, selumetinib was successfully developed and showed significant therapeutic effects on NF1 (Han et al., 2024).

Although some adverse reactions and cautions of selumetinib have been recorded in the package insert, including cardiac disorders, eye disorders, severe diarrhea, rash, and rhabdomyolysis, our study found some new potential ADEs based on real world data, such as hyperphosphataemia, menstrual disorder, blood bilirubin increased, haematuria and neutropenia, etc. Cardiac problems are one of the serious ADEs associated with selumetinib, and previous studies have shown that patients treated with selumetinib may have altered systolic function and reduced left ventricular ejection fraction (Caiffa et al., 2022; Gross et al., 2022). This study also detected cardiac valve disease, mitral valve incompetence, angina pectoris, pericardial effusion, ADEs that are not mentioned in the specification, which may help to deepen our understanding of the cardiotoxicity of selumetinib. Although the incidence of cardiotoxicity is low, it should also cause clinicians and patients to pay attention to avoid serious consequences. As reported previously, eye disorders including serous retinopathy, retinal detachment, blindness, and vision blurred, were another serious ADEs associated with selumetinib, and these ADEs were successfully detected in this study (Casey et al., 2021). Interestingly, many previous clinical studies of selumetinib reported its cardiac toxicity, and eye diseases were rarely mentioned (Dombi et al., 2016; Fangusaro et al., 2019; Gross et al., 2022). However, our study retrieved more ADE reports related to eye diseases than heart toxicity (28 vs. 16), which may be related to the population included in the clinical study. Blood creatine phosphokinase increased, skin-related ADEs, and gastrointestinal disorders were the most reported ADEs detected in this study, consistent with findings in clinical studies, where these ADEs were generally grade 1 and 2 and did not affect treatment, but some severe cases required drug discontinuation (Fangusaro et al., 2019; Hwang et al., 2022; Borgia et al., 2024).

Among the 53 ADE signals detected in this study, 33 had been mentioned or were related to the ADEs mentioned in the package insert, almost covering the ADEs included in the package insert. These results indirectly reflected the reliability of the results of this study. It should be noted that 20 new ADE signals were also found in this study, including hyperphosphataemia, pharyngitis streptococcal, blood bilirubin increased, haematuria, neutropenia, etc. After anti-tumor therapy, a large number of tumor cells die, and the rapid release of intracellular phosphorus into the blood resulting in the elevation of blood phosphorus may help to explain this phenomenon (Barbar et al., 2021). Rhabdomyolysis and decreased excretion of phosphorus in renal injury may also contribute to the elevation of blood phosphorus (Vervloet and van Ballegooijen, 2018). Since selumetinib has an adverse reaction of rhabdomyolysis, this study also found that renal injury may be a potential ADE signal of selumetinib, so hyperphosphatemia may be a secondary response to its toxic effects, which can also be understood as hyperphosphatemia may be a potential ADE of selumetinib. Pharyngitis streptococcal has not been reported in previous studies, but stomatitis has been reported and documented in the package insert, and studies have reported cases of discontinuation due to intolerable mucositis (Gross et al., 2023), and it is unclear whether pharyngitis is related to stomatitis induced by selumetinib. Blood bilirubin increased was not mentioned in the package insert, but package insert and clinical studies have pointed out that selumetinib can increase aspartate aminotransferase (AST) and alanine aminotransferase (ALT), suggesting its effect on liver function, therefore blood bilirubin increased may be related to liver injury (Fangusaro et al., 2021). In addition, anaemia, lymphopenia, and neutropenia have also been observed in clinical studies of selumetinib in the treatment of NF1, and these adverse reactions are mostly grade 1-2 (Fangusaro et al., 2021). The skin toxicity of selumetinib has been widely reported in the package insert and in previous studies (Gross et al., 2022; Borgia et al., 2024). Notably, the skin toxicity of selumetinib is not limited to rashes, peeling, and blisters, but also includes ingrowing nail, hair colour changes, hair texture abnormal, and alopecia, which also enriches our knowledge of its skin toxicity. Renal and urinary disorders was the SOC that possessed the highest number of reports among the new potential ADEs detected in this study, including proteinuria, pollakiuria, dysuria, and haematuria. Pollakiuria and dysuria may be associated with complications of NF1, which may lead to bladder dysfunction (Gross et al., 2020), while further studies are needed to confirm whether haematuria and proteinuria are caused by selumetinib. Dysarthria was also detected in this study, which may be associated with NF1-related airway compromise rather than selumetinib use (Gross et al., 2020). It has been suggested that personality traits may change in patients with tumors (Chow et al., 2020). The personality changes detected in this study might be related to the patients’ long-term suffering from the disease. There are also some undetected ADE signals, but reported by previous studies, such as hyponatremia, weight gain, serum amylase increased, and hyperuricemia, which may be related to underreporting of data (Gross et al., 2020; Fangusaro et al., 2021).

### 4.2 Analysis of potential toxicological mechanisms of ADE

On the basis of the understanding of selumetinib ADE, the toxicological mechanism of selumetinib was explored, involving the ADE signals that have been documented in the package insert and the ADE signals new identified in this study. PPI analysis reflected the possible toxicity targets involved in SOC of interest, among which EGFR, STAT3, AKT1, IL6 and BCL2 were at key nodes in the PPI network, suggesting their important contribution to selumetinib toxicity. Further mechanistic analysis revealed that the potential toxic targets of selumetinib were involved in a broad and complex mechanism, and the key pathways included PI3K pathway, apoptosis, ErbB signaling pathway, and EGFR tyrosine kinase inhibitor resistance.

PI3K/AKT pathway is one of the most important mechanisms regulating cell proliferation and differentiation, which is often over-activated in cancers, and anti-cancer strategies based on PI3K inhibitors have been widely studied and have shown good prospects (Belli et al., 2023). However, the side effects of PI3K inhibitors are also obvious, including hyperglycemia, rash, diarrhea, nausea and fatigue, which are also seen in selumetinib (Nunnery and Mayer, 2019). Virtual molecular docking is a common technique used in drug research to rapidly assess the binding between small molecules and proteins (Aziz et al., 2023a; Aziz et al., 2023b). In this study, the affinity of selumetinib with PI3K subunits PIK3CA, PIK3CD and PIK3R1 was tested by molecular docking technology, and selumetinib was found to bind freely to three PI3K subunits, although the affinity was weak, which may explain the mechanism of selumetinib causing rash, diarrhea, nausea and fatigue. As a MEK inhibitor, selumetinib can induce cancer cell apoptosis, which is the core mechanism of its treatment of NF1. However, the inhibitory effect on MEK in normal cells leads to its toxicity, and apoptosis in normal cells may help explain the multiple ADE of selumetinib. ErbB receptor family is also known as epidermal growth factor receptor (EGFR) family or type I receptor family. Activated ErbB receptors stimulate the activation of many downstream signaling pathways, including RAS/RAF/MEK/ERK, PI3K/AKT/TOR, PLCγ1, JAK/STAT, and SRC pathways (Wang, 2017; Hassan and Seno, 2022). ErbB receptors, especially EGFR and ErbB2 have been the primary choices as targets for developing cancer therapies. In fact, many EGFR tyrosine kinase inhibitors (EGFR-TKIs) have been approved for the treatment of cancers, showing good anti-cancer effect. However, there are also many side effects that cannot be ignored, such as skin toxicity, gastrointestinal toxicity and lung toxicity (Beech et al., 2018; Zhao et al., 2021). The main causes of toxic effects are off-target effects of drugs and multiple regulatory effects of target proteins under physiological and pathological conditions. These signaling pathways may contribute to selumetinib treatment of NF1, and may also be involved in selumetinib toxicity.

### 4.3 Shortcomings and limitations

This study also has some shortcomings and limitations: (1) FAERS, as a spontaneous reporting system, may have omissions, duplications, incomplete reports, and misreporting due to the varying knowledge backgrounds of those submitting the reports, which may have an impact on the results of this study. (2) The FAERS-based pharmacovigilance study is only a descriptive study, and the causal relationship between drugs and ADE cannot be determined, which is also the common problem of FAERS-based pharmacovigilance studies (Ni et al., 2023; Xiong et al., 2023). (3) Although the ADE signals detected in this study may be related to the administration of selumetinib, the patients’ co-morbidities status, and co-administration were not strictly differentiated. (4) In network toxicology, target prediction for selumetinib is highly dependent on database algorithms, which are in most cases experimentally unproven, and it is not clear whether the regulatory effect of drugs on the predicted targets is activation or inhibition.

## 5 Conclusion

In conclusion, this study investigated the ADE of selumetinib and identified some new potential ADE signals by pharmacovigilance method, and preliminarily revealed the toxicological mechanism of selumetinib ADE by network toxicology method, which provided a reference for a comprehensive and profound understanding of the safety of selumetinib. In addition, the ideas of this study also provide a new paradigm for the rapid discovery and analysis of the safety and toxicological mechanisms of newly marketed drugs.

## Data Availability

The original contributions presented in the study are included in the article/Supplementary Material, further inquiries can be directed to the corresponding authors.
