# Supplementary material for: Efficient analysis of adverse drug events and toxicological mechanisms of newly marketed drugs by integrating pharmacovigilance and network toxicology: selumetinib as an example
Source: Front Pharmacol. 2024 Aug 13;15:1432759. doi: 10.3389/fphar.2024.1432759 (PMC11347787; doi:10.3389/fphar.2024.1432759)
Supplement: Supplementary file 1 [file Table1.docx]

Supplementary Material

**Efficient analysis of adverse drug events and toxicological mechanisms of newly marketed drugs by integrating pharmacovigilance and network toxicology: selumetinib as an example**

Rui Xiong ^1, †^, Jing Lei ^2, †^, Lu Wang ^1, †^, Shipeng Zhang ^1^, Hengxu Liu ^1^, Hongping Wang ^3^, Tao Liu ^4, *^, Xiaodan Lai ^1, *^

^1^ Department of Pharmacy, Jiangbei Campus of The First Affiliated Hospital of Army Medical University (The 958th Hospital of Chinese People's Liberation Army), Chongqing, China; xiongrui@tmmu.edu.cn (R.X.), 499631970@qq.com (L.W.). Jasper-zsp@outlook.com (S.Z.), liuhengxu81@163.com (H.L.), laixiaodan0926@sina.com (X.L.)

^2^ Department of Pharmacy, Daping Hospital, Army Medical University, Chongqing, China; leij551@tmmu.edu.cn

^3^ Department of Pharmacy, The First Affiliated Hospital of Army Medical University, Chongqing, China. whp@tmmu.edu.cn.

^4^ Department of Infectious Diseases, Navy No.971 Hospital, Qingdao, Shandong Province, China; liu.tao@aliyun.com

^*^ Corresponding author: Tao Liu: liu.tao@aliyun.com, Xiaodan Lai: laixiaodan0926@sina.com

^†^ The authors contributed equally to the article.

Table S1: The number of ADE reports and signal detection results of selumetinib extracted from FAERS

| No. | PT | *a* | *b* | *c* | *d* | ROR (95% CI) | PRR (χ^2^） |
| --- | --- | --- | --- | --- | --- | --- | --- |
| 1 | Serous retinopathy | 4 | 511 | 63 | 4677893 | 581.23 (210.78, 1602.78) | 576.72 (2161.69) |
| 2 | Paronychia | 20 | 495 | 770 | 4677186 | 245.42 (156.09, 385.90) | 235.93 (4561.06) |
| 3 | Dermatitis acneiform | 24 | 491 | 1064 | 4676892 | 214.85 (142.00, 325.09) | 204.89 (4763.15) |
| 4 | Blood creatine phosphokinase increased | 52 | 463 | 3703 | 4674253 | 141.77 (106.24, 189.17) | 127.56 (6444.04) |
| 5 | Ingrowing nail | 6 | 509 | 409 | 4677547 | 134.81 (59.93, 303.25) | 133.25 (776.25) |
| 6 | Hyperphosphataemia | 4 | 511 | 342 | 4677614 | 107.06 (39.80, 287.99) | 106.24 (412.20) |
| 7 | Subretinal fluid | 3 | 512 | 344 | 4677612 | 79.67 (25.49, 249.08) | 79.22 (229.70) |
| 8 | Otitis media | 3 | 512 | 404 | 4677552 | 67.84 (21.72, 211.93) | 67.45 (194.97) |
| 9 | Retinal detachment | 10 | 505 | 1636 | 4676320 | 56.60 (30.21, 106.04) | 55.52 (532.34) |
| 10 | Hair colour changes | 12 | 503 | 2267 | 4675689 | 49.20 (27.72, 87.36) | 48.08 (550.58) |
| 11 | Ejection fraction decreased | 13 | 502 | 3607 | 4674349 | 33.56 (19.33, 58.26) | 32.74 (398.86) |
| 12 | Skin toxicity | 4 | 511 | 1310 | 4676646 | 27.94 (10.43, 74.85) | 27.74 (102.80) |
| 13 | Cardiac valve disease | 3 | 512 | 996 | 4676960 | 27.51 (8.83, 85.74) | 27.36 (75.98) |
| 14 | Menstrual disorder | 3 | 512 | 1216 | 4676740 | 22.54 (7.23, 70.20) | 22.41 (61.23) |
| 15 | Acne | 15 | 500 | 7049 | 4670907 | 19.88 (11.89, 33.24) | 19.33 (260.55) |
| 16 | Mitral valve incompetence | 3 | 512 | 1383 | 4676573 | 19.81 (6.36, 61.71) | 19.70 (53.16) |
| 17 | Personality change | 3 | 512 | 1436 | 4676520 | 19.08 (6.13, 59.43) | 18.98 (51.00) |
| 18 | Rash maculo-papular | 7 | 508 | 3891 | 4674065 | 16.55 (7.85, 34.92) | 16.34 (100.72) |
| 19 | Dermatitis | 6 | 509 | 3528 | 4674428 | 15.62 (6.98, 34.95) | 15.45 (81.00) |
| 20 | Cardiotoxicity | 4 | 511 | 2448 | 4675508 | 14.95 (5.59, 40.02) | 14.84 (51.58) |
| 21 | Pharyngitis streptococcal | 3 | 512 | 2053 | 4675903 | 13.35 (4.29, 41.55) | 13.27 (34.01) |
| 22 | Hair texture abnormal | 3 | 512 | 2068 | 4675888 | 13.25 (4.26, 41.25) | 13.18 (33.72) |
| 23 | Aspartate aminotransferase increased | 9 | 506 | 6532 | 4671424 | 12.72 (6.58, 24.60) | 12.52 (95.36) |
| 24 | Stomatitis | 12 | 503 | 11333 | 4666623 | 9.82 (5.54, 17.42) | 9.62 (92.79) |
| 25 | Proteinuria | 4 | 511 | 4047 | 4673909 | 9.04 (3.38, 24.19) | 8.98 (28.35) |
| 26 | Impaired healing | 4 | 511 | 4231 | 4673725 | 8.65 (3.23, 23.14) | 8.59 (26.81) |
| 27 | Blood bilirubin increased | 3 | 512 | 3443 | 4674513 | 7.96 (2.56, 24.76) | 7.91 (18.12) |
| 28 | Mouth ulceration | 3 | 512 | 3753 | 4674203 | 7.30 (2.34, 22.71) | 7.26 (16.20) |
| 29 | Angina pectoris | 3 | 512 | 3813 | 4674143 | 7.18 (2.31, 22.35) | 7.15 (15.86) |
| 30 | Pericardial effusion | 3 | 512 | 4114 | 4673842 | 6.66 (2.14, 20.72) | 6.62 (14.33) |
| 31 | Pollakiuria | 5 | 510 | 6925 | 4671031 | 6.61 (2.74, 15.96) | 6.56 (23.57) |
| 32 | Mucosal inflammation | 3 | 512 | 4162 | 4673794 | 6.58 (2.11, 20.48) | 6.55 (14.10) |
| 33 | Dysuria | 4 | 511 | 5630 | 4672326 | 6.50 (2.43, 17.38) | 6.45 (18.44) |
| 34 | Skin disorder | 5 | 510 | 7494 | 4670462 | 6.11 (2.53, 14.75) | 6.06 (21.15) |
| 35 | Eczema | 8 | 507 | 12084 | 4665872 | 6.09 (3.03, 12.25) | 6.01 (33.50) |
| 36 | Alopecia | 21 | 494 | 35005 | 4642951 | 5.64 (3.64, 8.73) | 5.45 (76.82) |
| 37 | Haematuria | 3 | 512 | 5068 | 4672888 | 5.40 (1.74, 16.81) | 5.38 (10.69) |
| 38 | Dry skin | 11 | 504 | 19920 | 4658036 | 5.10 (2.81, 9.28) | 5.02 (35.50) |
| 39 | Dysarthria | 3 | 512 | 5577 | 4672379 | 4.91 (1.58, 15.28) | 4.89 (9.28) |
| 40 | Oedema | 4 | 511 | 7685 | 4670271 | 4.76 (1.78, 12.73) | 4.73 (11.77) |
| 41 | Rash | 40 | 475 | 83419 | 4594537 | 4.64 (3.36, 6.40) | 4.36 (105.23) |
| 42 | Rhabdomyolysis | 3 | 512 | 6424 | 4671532 | 4.26 (1.37, 13.26) | 4.24 (7.44) |
| 43 | Abdominal pain | 17 | 498 | 37599 | 4640357 | 4.21 (2.60, 6.83) | 4.11 (40.26) |
| 44 | Blindness | 4 | 511 | 8699 | 4669257 | 4.20 (1.57, 11.24) | 4.18 (9.68) |
| 45 | Oedema peripheral | 6 | 509 | 13232 | 4664724 | 4.16 (1.86, 9.30) | 4.12 (14.20) |
| 46 | Muscular weakness | 7 | 508 | 16694 | 4661262 | 3.85 (1.82, 8.11) | 3.81 (14.55) |
| 47 | Epistaxis | 5 | 510 | 11933 | 4666023 | 3.83 (1.59, 9.25) | 3.81 (10.37) |
| 48 | Vision blurred | 7 | 508 | 23530 | 4654426 | 2.73 (1.29, 5.75) | 2.70 (7.54) |
| 49 | Sepsis | 5 | 510 | 17557 | 4660399 | 2.60 (1.08, 6.28) | 2.59 (4.88) |
| 50 | Myalgia | 7 | 508 | 25365 | 4652591 | 2.53 (1.20, 5.33) | 2.51 (6.37) |
| 51 | Anaemia | 8 | 507 | 30275 | 4647681 | 2.42 (1.20, 4.87) | 2.40 (6.58) |
| 52 | Diarrhoea | 28 | 487 | 124457 | 4553499 | 2.10 (1.44, 3.08) | 2.04 (15.33) |
| 53 | Neutropenia | 8 | 507 | 35448 | 4642508 | 2.07 (1.03, 4.16) | 2.05 (4.33) |
| 54 | Blood creatine phosphokinase MB | 1 | 514 | 2 | 4677954 | 4550.54 (411.97, 50264.65) | 4541.70 (3026.47) |
| 55 | Eczema vesicular | 1 | 514 | 6 | 4677950 | 1516.85 (182.29, 12621.97) | 1513.90 (1295.92) |
| 56 | Thrombosed varicose vein | 1 | 514 | 8 | 4677948 | 1137.63 (142.03, 9112.44) | 1135.43 (1007.49) |
| 57 | Mucocutaneous toxicity | 1 | 514 | 9 | 4677947 | 1011.23 (127.88, 7996.44) | 1009.27 (906.54) |
| 58 | Brain radiation necrosis | 1 | 514 | 9 | 4677947 | 1011.23 (127.88, 7996.44) | 1009.27 (906.54) |
| 59 | Urachal abnormality | 1 | 514 | 11 | 4677945 | 827.37 (106.62, 6420.36) | 825.76 (755.12) |
| 60 | Streptobacillus infection | 1 | 514 | 11 | 4677945 | 827.37 (106.62, 6420.36) | 825.76 (755.12) |
| 61 | Ovarian adenoma | 1 | 514 | 14 | 4677942 | 650.08 (85.32, 4952.87) | 648.81 (603.70) |
| 62 | Corneal irritation | 1 | 514 | 15 | 4677941 | 606.74 (80.00, 4601.88) | 605.56 (565.84) |
| 63 | Nail bed infection | 2 | 513 | 32 | 4677924 | 569.92 (136.22, 2384.40) | 567.71 (1064.88) |
| 64 | Blood growth hormone abnormal | 1 | 514 | 17 | 4677939 | 535.36 (71.11, 4030.34) | 534.32 (502.75) |
| 65 | Malignant glioma | 1 | 514 | 20 | 4677936 | 455.05 (60.96, 3397.09) | 454.17 (430.64) |
| 66 | Carbuncle | 1 | 514 | 28 | 4677928 | 325.04 (44.14, 2393.48) | 324.41 (311.29) |
| 67 | Urticaria papular | 1 | 514 | 30 | 4677926 | 303.37 (41.29, 2228.80) | 302.78 (291.08) |
| 68 | Blood alkaline phosphatase | 1 | 514 | 31 | 4677925 | 293.58 (40.00, 2154.67) | 293.01 (281.92) |
| 69 | Nodular rash | 1 | 514 | 31 | 4677925 | 293.58 (40.00, 2154.67) | 293.01 (281.92) |
| 70 | Genital candidiasis | 1 | 514 | 33 | 4677923 | 275.79 (37.65, 2020.27) | 275.25 (265.22) |
| 71 | Arachnoid cyst | 1 | 514 | 35 | 4677921 | 260.03 (35.56, 1901.65) | 259.53 (250.38) |
| 72 | Craniopharyngioma | 1 | 514 | 36 | 4677920 | 252.81 (34.59, 1847.41) | 252.32 (243.56) |
| 73 | Vulvitis | 1 | 514 | 37 | 4677919 | 245.97 (33.68, 1796.17) | 245.50 (237.10) |
| 74 | Optic glioma | 1 | 514 | 42 | 4677914 | 216.69 (29.77, 1577.43) | 216.27 (209.30) |
| 75 | Duodenal obstruction | 1 | 514 | 43 | 4677913 | 211.65 (29.09, 1539.92) | 211.24 (204.49) |
| 76 | Eczema infected | 1 | 514 | 44 | 4677912 | 206.84 (28.44, 1504.15) | 206.44 (199.90) |
| 77 | Xeroderma | 1 | 514 | 49 | 4677907 | 185.73 (25.60, 1347.63) | 185.38 (179.72) |
| 78 | Haemoglobinuria | 1 | 514 | 50 | 4677906 | 182.02 (25.10, 1320.16) | 181.67 (176.15) |
| 79 | Retinal depigmentation | 1 | 514 | 53 | 4677903 | 171.72 (23.70, 1244.06) | 171.39 (166.26) |
| 80 | Malignant neoplasm of spinal cord | 1 | 514 | 56 | 4677900 | 162.52 (22.45, 1176.26) | 162.20 (157.40) |
| 81 | Peripheral nerve lesion | 1 | 514 | 58 | 4677898 | 156.91 (21.69, 1135.02) | 156.61 (152.00) |
| 82 | Scarlet fever | 1 | 514 | 59 | 4677897 | 154.25 (21.33, 1115.47) | 153.96 (149.43) |
| 83 | Eyelash changes | 1 | 514 | 64 | 4677892 | 142.20 (19.69, 1027.00) | 141.93 (137.78) |
| 84 | Visual acuity reduced transiently | 1 | 514 | 69 | 4677887 | 131.90 (18.28, 951.53) | 131.64 (127.80) |
| 85 | Mitral valve prolapse | 2 | 513 | 146 | 4677810 | 124.91 (30.86, 505.56) | 124.43 (241.58) |
| 86 | Rhegmatogenous retinal detachment | 1 | 514 | 73 | 4677883 | 124.67 (17.29, 898.70) | 124.43 (120.79) |
| 87 | Orbital oedema | 1 | 514 | 77 | 4677879 | 118.19 (16.41, 851.42) | 117.97 (114.49) |
| 88 | Penile erythema | 1 | 514 | 81 | 4677875 | 112.36 (15.61, 808.87) | 112.14 (108.81) |
| 89 | Adnexal torsion | 1 | 514 | 83 | 4677873 | 109.65 (15.24, 789.15) | 109.44 (106.17) |
| 90 | Molluscum contagiosum | 1 | 514 | 84 | 4677872 | 108.34 (15.06, 779.65) | 108.14 (104.90) |
| 91 | Rotavirus infection | 1 | 514 | 84 | 4677872 | 108.34 (15.06, 779.65) | 108.14 (104.90) |
| 92 | Musculoskeletal toxicity | 1 | 514 | 87 | 4677869 | 104.61 (14.54, 752.46) | 104.41 (101.25) |
| 93 | Pelvic neoplasm | 1 | 514 | 90 | 4677866 | 101.12 (14.06, 727.11) | 100.93 (97.85) |
| 94 | Precocious puberty | 1 | 514 | 95 | 4677861 | 95.80 (13.33, 688.45) | 95.61 (92.65) |
| 95 | Ocular toxicity | 2 | 513 | 197 | 4677759 | 92.57 (22.93, 373.77) | 92.22 (178.65) |
| 96 | Scrotal pain | 1 | 514 | 103 | 4677853 | 88.36 (12.31, 634.47) | 88.19 (85.37) |
| 97 | Malignant transformation | 1 | 514 | 110 | 4677846 | 82.74 (11.53, 593.73) | 82.58 (79.86) |
| 98 | Chorioretinopathy | 2 | 513 | 235 | 4677721 | 77.60 (19.24, 312.97) | 77.31 (149.37) |
| 99 | Nail infection | 2 | 513 | 236 | 4677720 | 77.27 (19.16, 311.64) | 76.98 (148.73) |
| 100 | Corneal perforation | 1 | 514 | 120 | 4677836 | 75.84 (10.58, 543.85) | 75.70 (73.10) |
| 101 | Soft tissue disorder | 1 | 514 | 120 | 4677836 | 75.84 (10.58, 543.85) | 75.70 (73.10) |
| 102 | Blood bilirubin decreased | 1 | 514 | 122 | 4677834 | 74.60 (10.40, 534.87) | 74.45 (71.88) |
| 103 | Retinal toxicity | 2 | 513 | 247 | 4677709 | 73.83 (18.31, 297.68) | 73.55 (141.98) |
| 104 | Vulvovaginal inflammation | 1 | 514 | 125 | 4677831 | 72.81 (10.16, 521.93) | 72.67 (70.12) |
| 105 | Glioma | 1 | 514 | 134 | 4677822 | 67.92 (9.48, 486.62) | 67.79 (65.32) |
| 106 | Cutaneous symptom | 1 | 514 | 142 | 4677814 | 64.09 (8.95, 459.01) | 63.97 (61.55) |
| 107 | Ventricular dysfunction | 2 | 513 | 291 | 4677665 | 62.67 (15.56, 252.45) | 62.43 (120.07) |
| 108 | Seborrhoeic dermatitis | 2 | 513 | 338 | 4677618 | 53.95 (13.40, 217.20) | 53.75 (102.93) |
| 109 | Mastoiditis | 1 | 514 | 175 | 4677781 | 52.00 (7.27, 371.97) | 51.91 (49.64) |
| 110 | Cell death | 2 | 513 | 360 | 4677596 | 50.66 (12.59, 203.88) | 50.46 (96.44) |
| 111 | Head and neck cancer | 1 | 514 | 181 | 4677775 | 50.28 (7.03, 359.57) | 50.18 (47.94) |
| 112 | Perioral dermatitis | 1 | 514 | 187 | 4677769 | 48.67 (6.81, 347.98) | 48.57 (46.35) |
| 113 | Adrenal gland cancer | 1 | 514 | 191 | 4677765 | 47.65 (6.66, 340.65) | 47.56 (45.34) |
| 114 | Optic nerve disorder | 1 | 514 | 192 | 4677764 | 47.40 (6.63, 338.87) | 47.31 (45.10) |
| 115 | Electrocardiogram T wave abnormal | 1 | 514 | 192 | 4677764 | 47.40 (6.63, 338.87) | 47.31 (45.10) |
| 116 | Otitis externa | 1 | 514 | 196 | 4677760 | 46.43 (6.50, 331.92) | 46.34 (44.14) |
| 117 | Conduction disorder | 1 | 514 | 202 | 4677754 | 45.05 (6.30, 322.01) | 44.97 (42.78) |
| 118 | Hand dermatitis | 1 | 514 | 211 | 4677745 | 43.13 (6.04, 308.21) | 43.05 (40.88) |
| 119 | Enzyme level increased | 1 | 514 | 212 | 4677744 | 42.93 (6.01, 306.75) | 42.85 (40.68) |
| 120 | Retinal oedema | 2 | 513 | 425 | 4677531 | 42.91 (10.67, 172.59) | 42.75 (81.16) |
| 121 | Endocrine disorder | 1 | 514 | 226 | 4677730 | 40.27 (5.64, 287.67) | 40.19 (38.05) |
| 122 | Arnold-Chiari malformation | 1 | 514 | 234 | 4677722 | 38.89 (5.44, 277.79) | 38.82 (36.69) |
| 123 | Neurodermatitis | 2 | 513 | 470 | 4677486 | 38.80 (9.65, 156.02) | 38.65 (73.05) |
| 124 | Lymphocyte count abnormal | 1 | 514 | 238 | 4677718 | 38.24 (5.35, 273.10) | 38.17 (36.04) |
| 125 | Conversion disorder | 1 | 514 | 239 | 4677717 | 38.08 (5.33, 271.95) | 38.01 (35.88) |
| 126 | Gastrointestinal bacterial overgrowth | 1 | 514 | 245 | 4677711 | 37.15 (5.20, 265.27) | 37.08 (34.96) |
| 127 | Neck mass | 2 | 513 | 496 | 4677460 | 36.77 (9.14, 147.82) | 36.63 (69.04) |
| 128 | Gastrointestinal stoma complication | 1 | 514 | 248 | 4677708 | 36.70 (5.14, 262.05) | 36.63 (34.52) |
| 129 | Tumour haemorrhage | 2 | 513 | 519 | 4677437 | 35.14 (8.74, 141.25) | 35.00 (65.82) |
| 130 | Onychomadesis | 2 | 513 | 544 | 4677412 | 33.52 (8.34, 134.74) | 33.39 (62.63) |
| 131 | Heart valve incompetence | 2 | 513 | 549 | 4677407 | 33.22 (8.26, 133.51) | 33.09 (62.02) |
| 132 | Skin hypopigmentation | 1 | 514 | 277 | 4677679 | 32.85 (4.60, 234.51) | 32.79 (30.71) |
| 133 | Superficial vein thrombosis | 1 | 514 | 289 | 4677667 | 31.49 (4.41, 224.74) | 31.43 (29.36) |
| 134 | Osteolysis | 1 | 514 | 292 | 4677664 | 31.17 (4.37, 222.42) | 31.11 (29.04) |
| 135 | High density lipoprotein decreased | 1 | 514 | 293 | 4677663 | 31.06 (4.35, 221.66) | 31.00 (28.94) |
| 136 | Polycythaemia | 1 | 514 | 299 | 4677657 | 30.44 (4.27, 217.20) | 30.38 (28.32) |
| 137 | Blood calcium abnormal | 1 | 514 | 311 | 4677645 | 29.26 (4.10, 208.79) | 29.21 (27.16) |
| 138 | Tympanic membrane perforation | 1 | 514 | 323 | 4677633 | 28.17 (3.95, 201.01) | 28.12 (26.08) |
| 139 | Paresis | 1 | 514 | 333 | 4677623 | 27.33 (3.83, 194.96) | 27.28 (25.24) |
| 140 | Blood phosphorus increased | 1 | 514 | 338 | 4677618 | 26.92 (3.77, 192.06) | 26.87 (24.84) |
| 141 | Rash pustular | 2 | 513 | 687 | 4677269 | 26.54 (6.61, 106.63) | 26.44 (48.83) |
| 142 | Thrombophlebitis | 1 | 514 | 356 | 4677600 | 25.56 (3.58, 182.33) | 25.52 (23.49) |
| 143 | Functional gastrointestinal disorder | 2 | 513 | 715 | 4677241 | 25.50 (6.35, 102.45) | 25.41 (46.77) |
| 144 | Sinus node dysfunction | 1 | 514 | 362 | 4677594 | 25.14 (3.52, 179.30) | 25.09 (23.07) |
| 145 | Cheilitis | 2 | 513 | 745 | 4677211 | 24.48 (6.09, 98.32) | 24.38 (44.74) |
| 146 | Hyperaemia | 1 | 514 | 376 | 4677580 | 24.20 (3.39, 172.60) | 24.16 (22.14) |
| 147 | Eyelid disorder | 1 | 514 | 377 | 4677579 | 24.14 (3.38, 172.14) | 24.09 (22.08) |
| 148 | Large intestinal obstruction | 1 | 514 | 385 | 4677571 | 23.64 (3.31, 168.56) | 23.59 (21.58) |
| 149 | Temporomandibular joint syndrome | 1 | 514 | 388 | 4677568 | 23.45 (3.29, 167.25) | 23.41 (21.40) |
| 150 | Duodenitis | 1 | 514 | 391 | 4677565 | 23.27 (3.26, 165.96) | 23.23 (21.22) |
| 151 | Hypernatraemia | 2 | 513 | 787 | 4677169 | 23.17 (5.77, 93.06) | 23.08 (42.15) |
| 152 | Viral upper respiratory tract infection | 1 | 514 | 397 | 4677559 | 22.92 (3.21, 163.45) | 22.88 (20.87) |
| 153 | Blood sodium increased | 1 | 514 | 410 | 4677546 | 22.20 (3.11, 158.25) | 22.15 (20.15) |
| 154 | Henoch-Schonlein purpura | 1 | 514 | 411 | 4677545 | 22.14 (3.11, 157.87) | 22.10 (20.10) |
| 155 | Hydrocephalus | 2 | 513 | 830 | 4677126 | 21.97 (5.47, 88.23) | 21.89 (39.78) |
| 156 | Shoulder fracture | 1 | 514 | 416 | 4677540 | 21.88 (3.07, 155.97) | 21.84 (19.84) |
| 157 | Strabismus | 1 | 514 | 432 | 4677524 | 21.07 (2.95, 150.18) | 21.03 (19.03) |
| 158 | Intra-abdominal fluid collection | 1 | 514 | 442 | 4677514 | 20.59 (2.89, 146.77) | 20.55 (18.56) |
| 159 | Ear swelling | 1 | 514 | 448 | 4677508 | 20.31 (2.85, 144.80) | 20.28 (18.29) |
| 160 | Ulcerative keratitis | 1 | 514 | 448 | 4677508 | 20.31 (2.85, 144.80) | 20.28 (18.29) |
| 161 | Tricuspid valve incompetence | 2 | 513 | 921 | 4677035 | 19.80 (4.93, 79.50) | 19.73 (35.48) |
| 162 | Corneal oedema | 1 | 514 | 460 | 4677496 | 19.78 (2.78, 141.01) | 19.75 (17.76) |
| 163 | Intracranial pressure increased | 2 | 513 | 964 | 4676992 | 18.91 (4.71, 75.94) | 18.85 (33.73) |
| 164 | Skin hyperpigmentation | 2 | 513 | 965 | 4676991 | 18.90 (4.71, 75.87) | 18.83 (33.69) |
| 165 | Venous thrombosis | 1 | 514 | 509 | 4677447 | 17.88 (2.51, 127.41) | 17.85 (15.87) |
| 166 | Blood potassium abnormal | 1 | 514 | 512 | 4677444 | 17.77 (2.49, 126.66) | 17.74 (15.77) |
| 167 | Aortic valve incompetence | 1 | 514 | 515 | 4677441 | 17.67 (2.48, 125.92) | 17.64 (15.67) |
| 168 | Chronic sinusitis | 1 | 514 | 529 | 4677427 | 17.20 (2.41, 122.58) | 17.17 (15.20) |
| 169 | Metastases to meninges | 1 | 514 | 536 | 4677420 | 16.98 (2.38, 120.98) | 16.95 (14.98) |
| 170 | Bundle branch block right | 1 | 514 | 538 | 4677418 | 16.91 (2.37, 120.53) | 16.88 (14.92) |
| 171 | Hordeolum | 2 | 513 | 1104 | 4676852 | 16.52 (4.11, 66.30) | 16.46 (28.99) |
| 172 | Oral mucosal blistering | 2 | 513 | 1131 | 4676825 | 16.12 (4.02, 64.72) | 16.06 (28.21) |
| 173 | Hair disorder | 2 | 513 | 1131 | 4676825 | 16.12 (4.02, 64.72) | 16.06 (28.21) |
| 174 | Polydipsia | 1 | 514 | 569 | 4677387 | 15.99 (2.24, 113.95) | 15.96 (14.00) |
| 175 | Hyperuricaemia | 1 | 514 | 597 | 4677359 | 15.24 (2.14, 108.60) | 15.22 (13.26) |
| 176 | Localised oedema | 1 | 514 | 626 | 4677330 | 14.54 (2.04, 103.56) | 14.51 (12.56) |
| 177 | Cervical vertebral fracture | 1 | 514 | 633 | 4677323 | 14.38 (2.02, 102.41) | 14.35 (12.40) |
| 178 | Cystitis haemorrhagic | 1 | 514 | 635 | 4677321 | 14.33 (2.01, 102.09) | 14.30 (12.36) |
| 179 | Eye injury | 1 | 514 | 637 | 4677319 | 14.29 (2.01, 101.77) | 14.26 (12.31) |
| 180 | Erythema nodosum | 1 | 514 | 656 | 4677300 | 13.87 (1.95, 98.81) | 13.85 (11.90) |
| 181 | Amylase increased | 1 | 514 | 675 | 4677281 | 13.48 (1.89, 96.03) | 13.46 (11.52) |
| 182 | Stress fracture | 1 | 514 | 676 | 4677280 | 13.46 (1.89, 95.89) | 13.44 (11.50) |
| 183 | Vaginal infection | 1 | 514 | 687 | 4677269 | 13.25 (1.86, 94.35) | 13.22 (11.28) |
| 184 | Monoplegia | 1 | 514 | 693 | 4677263 | 13.13 (1.84, 93.53) | 13.11 (11.17) |
| 185 | Muscle fatigue | 1 | 514 | 731 | 4677225 | 12.45 (1.75, 88.66) | 12.43 (10.49) |
| 186 | Gastrointestinal perforation | 1 | 514 | 785 | 4677171 | 11.59 (1.63, 82.55) | 11.57 (9.65) |
| 187 | Blood phosphorus decreased | 1 | 514 | 794 | 4677162 | 11.46 (1.61, 81.62) | 11.44 (9.52) |
| 188 | Rhinitis allergic | 1 | 514 | 813 | 4677143 | 11.19 (1.57, 79.71) | 11.17 (9.25) |
| 189 | Tibia fracture | 1 | 514 | 841 | 4677115 | 10.82 (1.52, 77.05) | 10.80 (8.88) |
| 190 | Haemoglobin increased | 1 | 514 | 856 | 4677100 | 10.63 (1.49, 75.70) | 10.61 (8.70) |
| 191 | Ear disorder | 1 | 514 | 866 | 4677090 | 10.51 (1.48, 74.82) | 10.49 (8.58) |
| 192 | Blood albumin decreased | 1 | 514 | 866 | 4677090 | 10.51 (1.48, 74.82) | 10.49 (8.58) |
| 193 | Pustule | 1 | 514 | 891 | 4677065 | 10.21 (1.43, 72.72) | 10.19 (8.28) |
| 194 | Visual field defect | 1 | 514 | 906 | 4677050 | 10.04 (1.41, 71.51) | 10.03 (8.12) |
| 195 | Dermatitis exfoliative generalised | 1 | 514 | 963 | 4676993 | 9.45 (1.33, 67.28) | 9.43 (7.53) |
| 196 | Streptococcal infection | 1 | 514 | 981 | 4676975 | 9.28 (1.30, 66.04) | 9.26 (7.36) |
| 197 | Lipase increased | 1 | 514 | 1024 | 4676932 | 8.89 (1.25, 63.26) | 8.87 (6.98) |
| 198 | Skin infection | 2 | 513 | 2104 | 4675852 | 8.66 (2.16, 34.76) | 8.63 (13.49) |
| 199 | Joint effusion | 1 | 514 | 1051 | 4676905 | 8.66 (1.22, 61.64) | 8.64 (6.75) |
| 200 | Eyelid oedema | 1 | 514 | 1056 | 4676900 | 8.62 (1.21, 61.34) | 8.60 (6.71) |
| 201 | Muscle injury | 1 | 514 | 1110 | 4676846 | 8.20 (1.15, 58.36) | 8.18 (6.30) |
| 202 | Hyperlipidaemia | 1 | 514 | 1110 | 4676846 | 8.20 (1.15, 58.36) | 8.18 (6.30) |
| 203 | Brain neoplasm | 2 | 513 | 2250 | 4675706 | 8.10 (2.02, 32.50) | 8.07 (12.39) |
| 204 | Skin reaction | 2 | 513 | 2268 | 4675688 | 8.04 (2.00, 32.24) | 8.01 (12.26) |
| 205 | Hypoalbuminaemia | 1 | 514 | 1150 | 4676806 | 7.91 (1.11, 56.33) | 7.90 (6.02) |
| 206 | Purpura | 1 | 514 | 1158 | 4676798 | 7.86 (1.10, 55.94) | 7.84 (5.97) |
| 207 | Mouth haemorrhage | 1 | 514 | 1186 | 4676770 | 7.67 (1.08, 54.61) | 7.66 (5.79) |
| 208 | Hemiplegia | 1 | 514 | 1189 | 4676767 | 7.65 (1.07, 54.48) | 7.64 (5.77) |
| 209 | Sudden death | 1 | 514 | 1190 | 4676766 | 7.65 (1.07, 54.43) | 7.63 (5.76) |
| 210 | Oral disorder | 1 | 514 | 1199 | 4676757 | 7.59 (1.07, 54.02) | 7.58 (5.70) |
| 211 | Osteomyelitis | 2 | 513 | 2414 | 4675542 | 7.55 (1.88, 30.29) | 7.53 (11.31) |
| 212 | Ovarian cyst | 1 | 514 | 1233 | 4676723 | 7.38 (1.04, 52.53) | 7.37 (5.50) |
| 213 | Furuncle | 1 | 514 | 1288 | 4676668 | 7.06 (0.99, 50.28) | 7.05 (5.19) |
| 214 | Hypotonia | 1 | 514 | 1297 | 4676659 | 7.02 (0.99, 49.94) | 7.00 (5.14) |
| 215 | Rhinitis | 1 | 514 | 1304 | 4676652 | 6.98 (0.98, 49.67) | 6.97 (5.11) |
| 216 | Cardiac dysfunction | 1 | 514 | 1349 | 4676607 | 6.74 (0.95, 48.01) | 6.73 (4.88) |
| 217 | Myopathy | 1 | 514 | 1379 | 4676577 | 6.60 (0.93, 46.96) | 6.59 (4.74) |
| 218 | Skin laceration | 2 | 513 | 2766 | 4675190 | 6.59 (1.64, 26.43) | 6.57 (9.44) |
| 219 | Nail disorder | 1 | 514 | 1387 | 4676569 | 6.56 (0.92, 46.69) | 6.55 (4.70) |
| 220 | Hand fracture | 1 | 514 | 1467 | 4676489 | 6.20 (0.87, 44.14) | 6.19 (4.35) |
| 221 | Abnormal faeces | 1 | 514 | 1503 | 4676453 | 6.05 (0.85, 43.08) | 6.04 (4.21) |
| 222 | Cholecystitis | 1 | 514 | 1537 | 4676419 | 5.92 (0.83, 42.13) | 5.91 (4.08) |
| 223 | Hypothermia | 1 | 514 | 1555 | 4676401 | 5.85 (0.82, 41.64) | 5.84 (4.01) |
| 224 | Affective disorder | 1 | 514 | 1560 | 4676396 | 5.83 (0.82, 41.51) | 5.82 (3.99) |
| 225 | Hypocalcaemia | 2 | 513 | 3156 | 4674800 | 5.77 (1.44, 23.16) | 5.76 (7.86) |
| 226 | Petechiae | 1 | 514 | 1587 | 4676369 | 5.73 (0.81, 40.80) | 5.72 (3.90) |
| 227 | Acute generalised exanthematous pustulosis | 1 | 514 | 1608 | 4676348 | 5.66 (0.79, 40.27) | 5.65 (3.82) |
| 228 | Folliculitis | 1 | 514 | 1637 | 4676319 | 5.56 (0.78, 39.55) | 5.55 (3.73) |
| 229 | Eyelid ptosis | 1 | 514 | 1639 | 4676317 | 5.55 (0.78, 39.51) | 5.54 (3.72) |
| 230 | Malnutrition | 1 | 514 | 1656 | 4676300 | 5.49 (0.77, 39.10) | 5.49 (3.67) |
| 231 | Hyperbilirubinaemia | 1 | 514 | 1693 | 4676263 | 5.37 (0.76, 38.25) | 5.37 (3.55) |
| 232 | Ataxia | 1 | 514 | 1751 | 4676205 | 5.20 (0.73, 36.98) | 5.19 (3.38) |
| 233 | Micturition urgency | 1 | 514 | 1760 | 4676196 | 5.17 (0.73, 36.79) | 5.16 (3.35) |
| 234 | Hypertensive crisis | 1 | 514 | 1775 | 4676181 | 5.13 (0.72, 36.48) | 5.12 (3.31) |
| 235 | Blood lactate dehydrogenase increased | 1 | 514 | 1780 | 4676176 | 5.11 (0.72, 36.37) | 5.10 (3.30) |
| 236 | Bladder disorder | 1 | 514 | 1780 | 4676176 | 5.11 (0.72, 36.37) | 5.10 (3.30) |
| 237 | Dry throat | 1 | 514 | 1794 | 4676162 | 5.07 (0.71, 36.09) | 5.06 (3.26) |
| 238 | Dermatitis contact | 1 | 514 | 1803 | 4676153 | 5.05 (0.71, 35.91) | 5.04 (3.24) |
| 239 | Peritonitis | 1 | 514 | 1818 | 4676138 | 5.00 (0.70, 35.61) | 5.00 (3.20) |
| 240 | Presyncope | 2 | 513 | 3723 | 4674233 | 4.89 (1.22, 19.63) | 4.88 (6.17) |
| 241 | Dizziness postural | 1 | 514 | 1954 | 4676002 | 4.66 (0.65, 33.13) | 4.65 (2.86) |
| 242 | Thirst | 2 | 513 | 3916 | 4674040 | 4.65 (1.16, 18.66) | 4.64 (5.71) |
| 243 | Metastases to bone | 2 | 513 | 3948 | 4674008 | 4.62 (1.15, 18.51) | 4.60 (5.64) |
| 244 | Hypertransaminasaemia | 1 | 514 | 1982 | 4675974 | 4.59 (0.64, 32.66) | 4.58 (2.80) |
| 245 | Aphthous ulcer | 1 | 514 | 1996 | 4675960 | 4.56 (0.64, 32.43) | 4.55 (2.77) |
| 246 | International normalised ratio increased | 1 | 514 | 2002 | 4675954 | 4.54 (0.64, 32.34) | 4.54 (2.76) |
| 247 | Facial paralysis | 1 | 514 | 2007 | 4675949 | 4.53 (0.64, 32.26) | 4.53 (2.75) |
| 248 | Brain oedema | 1 | 514 | 2016 | 4675940 | 4.51 (0.63, 32.11) | 4.51 (2.73) |
| 249 | Skin irritation | 2 | 513 | 4039 | 4673917 | 4.51 (1.12, 18.09) | 4.50 (5.44) |
| 250 | Tendonitis | 1 | 514 | 2025 | 4675931 | 4.49 (0.63, 31.97) | 4.49 (2.71) |
| 251 | Haematotoxicity | 1 | 514 | 2065 | 4675891 | 4.41 (0.62, 31.35) | 4.40 (2.63) |
| 252 | Eosinophil count increased | 1 | 514 | 2068 | 4675888 | 4.40 (0.62, 31.30) | 4.39 (2.62) |
| 253 | Anal incontinence | 1 | 514 | 2103 | 4675853 | 4.33 (0.61, 30.78) | 4.32 (2.55) |
| 254 | Hepatitis | 2 | 513 | 4215 | 4673741 | 4.32 (1.08, 17.34) | 4.31 (5.09) |
| 255 | Oral pain | 2 | 513 | 4224 | 4673732 | 4.31 (1.08, 17.30) | 4.30 (5.07) |
| 256 | Body temperature decreased | 1 | 514 | 2112 | 4675844 | 4.31 (0.61, 30.65) | 4.30 (2.53) |
| 257 | Psychomotor hyperactivity | 1 | 514 | 2207 | 4675749 | 4.12 (0.58, 29.33) | 4.12 (2.36) |
| 258 | Cardiomyopathy | 1 | 514 | 2217 | 4675739 | 4.10 (0.58, 29.20) | 4.10 (2.34) |
| 259 | Cyst | 1 | 514 | 2240 | 4675716 | 4.06 (0.57, 28.90) | 4.06 (2.30) |
| 260 | Sinus tachycardia | 1 | 514 | 2260 | 4675696 | 4.03 (0.57, 28.64) | 4.02 (2.27) |
| 261 | Dermatitis allergic | 1 | 514 | 2267 | 4675689 | 4.01 (0.56, 28.55) | 4.01 (2.26) |
| 262 | Visual acuity reduced | 1 | 514 | 2333 | 4675623 | 3.90 (0.55, 27.75) | 3.89 (2.15) |
| 263 | Localised infection | 2 | 513 | 4730 | 4673226 | 3.85 (0.96, 15.45) | 3.84 (4.20) |
| 264 | Respiratory tract congestion | 1 | 514 | 2366 | 4675590 | 3.84 (0.54, 27.36) | 3.84 (2.10) |
| 265 | Attention deficit hyperactivity disorder | 1 | 514 | 2396 | 4675560 | 3.80 (0.53, 27.02) | 3.79 (2.06) |
| 266 | Respiratory depression | 1 | 514 | 2415 | 4675541 | 3.77 (0.53, 26.80) | 3.76 (2.03) |
| 267 | Mass | 1 | 514 | 2425 | 4675531 | 3.75 (0.53, 26.69) | 3.75 (2.01) |
| 268 | Quality of life decreased | 1 | 514 | 2434 | 4675522 | 3.74 (0.53, 26.59) | 3.73 (2.00) |
| 269 | Musculoskeletal chest pain | 1 | 514 | 2435 | 4675521 | 3.74 (0.52, 26.58) | 3.73 (2.00) |
| 270 | Paralysis | 1 | 514 | 2453 | 4675503 | 3.71 (0.52, 26.39) | 3.70 (1.97) |
| 271 | Abnormal dreams | 1 | 514 | 2471 | 4675485 | 3.68 (0.52, 26.19) | 3.68 (1.95) |
| 272 | Blindness unilateral | 1 | 514 | 2502 | 4675454 | 3.64 (0.51, 25.87) | 3.63 (1.91) |
| 273 | Tooth injury | 1 | 514 | 2505 | 4675451 | 3.63 (0.51, 25.84) | 3.63 (1.90) |
| 274 | Blood alkaline phosphatase increased | 1 | 514 | 2527 | 4675429 | 3.60 (0.51, 25.61) | 3.59 (1.87) |
| 275 | Liver function test increased | 2 | 513 | 5074 | 4672882 | 3.59 (0.90, 14.40) | 3.58 (3.72) |
| 276 | Rash papular | 1 | 514 | 2538 | 4675418 | 3.58 (0.50, 25.50) | 3.58 (1.86) |
| 277 | Cardio-respiratory arrest | 2 | 513 | 5165 | 4672791 | 3.53 (0.88, 14.15) | 3.52 (3.61) |
| 278 | Haemorrhoids | 1 | 514 | 2614 | 4675342 | 3.48 (0.49, 24.76) | 3.47 (1.76) |
| 279 | Hyperkalaemia | 2 | 513 | 5402 | 4672554 | 3.37 (0.84, 13.52) | 3.36 (3.32) |
| 280 | Skin lesion | 2 | 513 | 5442 | 4672514 | 3.35 (0.83, 13.42) | 3.34 (3.28) |
| 281 | Ulcer | 1 | 514 | 2722 | 4675234 | 3.34 (0.47, 23.78) | 3.34 (1.64) |
| 282 | Sciatica | 1 | 514 | 2736 | 4675220 | 3.32 (0.47, 23.66) | 3.32 (1.62) |
| 283 | Obstructive airways disorder | 1 | 514 | 2762 | 4675194 | 3.29 (0.46, 23.43) | 3.29 (1.59) |
| 284 | Gastric ulcer | 1 | 514 | 2772 | 4675184 | 3.28 (0.46, 23.35) | 3.28 (1.58) |
| 285 | Pneumothorax | 1 | 514 | 2880 | 4675076 | 3.16 (0.44, 22.47) | 3.15 (1.47) |
| 286 | Pericarditis | 1 | 514 | 2892 | 4675064 | 3.15 (0.44, 22.38) | 3.14 (1.46) |
| 287 | Abscess | 1 | 514 | 2916 | 4675040 | 3.12 (0.44, 22.19) | 3.12 (1.44) |
| 288 | Irritable bowel syndrome | 1 | 514 | 2933 | 4675023 | 3.10 (0.44, 22.06) | 3.10 (1.42) |
| 289 | Epilepsy | 2 | 513 | 5890 | 4672066 | 3.09 (0.77, 12.40) | 3.08 (2.82) |
| 290 | Photosensitivity reaction | 1 | 514 | 2946 | 4675010 | 3.09 (0.43, 21.97) | 3.08 (1.41) |
| 291 | Dyskinesia | 2 | 513 | 6013 | 4671943 | 3.03 (0.76, 12.15) | 3.02 (2.71) |
| 292 | Blister | 3 | 512 | 9072 | 4668884 | 3.02 (0.97, 9.38) | 3.00 (4.02) |
| 293 | Rash macular | 2 | 513 | 6136 | 4671820 | 2.97 (0.74, 11.90) | 2.96 (2.60) |
| 294 | Photophobia | 1 | 514 | 3065 | 4674891 | 2.97 (0.42, 21.11) | 2.96 (1.30) |
| 295 | Intervertebral disc protrusion | 1 | 514 | 3090 | 4674866 | 2.94 (0.41, 20.94) | 2.94 (1.28) |
| 296 | Rash pruritic | 3 | 512 | 9297 | 4668659 | 2.94 (0.95, 9.16) | 2.93 (3.82) |
| 297 | Musculoskeletal discomfort | 1 | 514 | 3285 | 4674671 | 2.77 (0.39, 19.70) | 2.77 (1.13) |
| 298 | Muscle twitching | 1 | 514 | 3299 | 4674657 | 2.76 (0.39, 19.61) | 2.75 (1.12) |
| 299 | Nervous system disorder | 1 | 514 | 3322 | 4674634 | 2.74 (0.38, 19.48) | 2.73 (1.10) |
| 300 | Gastrointestinal haemorrhage | 3 | 512 | 10204 | 4667752 | 2.68 (0.86, 8.34) | 2.67 (3.14) |
| 301 | Multiple organ dysfunction syndrome | 2 | 513 | 6823 | 4671133 | 2.67 (0.67, 10.70) | 2.66 (2.08) |
| 302 | Bacterial infection | 1 | 514 | 3411 | 4674545 | 2.67 (0.37, 18.97) | 2.66 (1.04) |
| 303 | Respiratory failure | 3 | 512 | 10574 | 4667382 | 2.59 (0.83, 8.05) | 2.58 (2.90) |
| 304 | Foot fracture | 1 | 514 | 3517 | 4674439 | 2.59 (0.36, 18.40) | 2.58 (0.97) |
| 305 | Melaena | 1 | 514 | 3529 | 4674427 | 2.58 (0.36, 18.33) | 2.57 (0.96) |
| 306 | Colitis | 2 | 513 | 7315 | 4670641 | 2.49 (0.62, 9.98) | 2.48 (1.77) |
| 307 | Musculoskeletal pain | 1 | 514 | 3655 | 4674301 | 2.49 (0.35, 17.70) | 2.49 (0.89) |
| 308 | Haematoma | 1 | 514 | 3667 | 4674289 | 2.48 (0.35, 17.64) | 2.48 (0.88) |
| 309 | Type 2 diabetes mellitus | 1 | 514 | 3672 | 4674284 | 2.48 (0.35, 17.62) | 2.47 (0.88) |
| 310 | Upper limb fracture | 1 | 514 | 3702 | 4674254 | 2.46 (0.35, 17.48) | 2.45 (0.86) |
| 311 | Sinus disorder | 1 | 514 | 3752 | 4674204 | 2.42 (0.34, 17.24) | 2.42 (0.83) |
| 312 | Gastritis | 1 | 514 | 3771 | 4674185 | 2.41 (0.34, 17.16) | 2.41 (0.82) |
| 313 | Transaminases increased | 1 | 514 | 3777 | 4674179 | 2.41 (0.34, 17.13) | 2.40 (0.82) |
| 314 | Cytopenia | 1 | 514 | 3842 | 4674114 | 2.37 (0.33, 16.84) | 2.36 (0.79) |
| 315 | Irritability | 2 | 513 | 7805 | 4670151 | 2.33 (0.58, 9.35) | 2.33 (1.52) |
| 316 | Kidney infection | 1 | 514 | 4000 | 4673956 | 2.27 (0.32, 16.17) | 2.27 (0.71) |
| 317 | Skin fissures | 1 | 514 | 4005 | 4673951 | 2.27 (0.32, 16.15) | 2.27 (0.71) |
| 318 | Femur fracture | 1 | 514 | 4027 | 4673929 | 2.26 (0.32, 16.07) | 2.26 (0.70) |
| 319 | Dysphagia | 4 | 511 | 16303 | 4661653 | 2.24 (0.84, 5.99) | 2.23 (2.72) |
| 320 | Lymphocyte count decreased | 1 | 514 | 4087 | 4673869 | 2.22 (0.31, 15.83) | 2.22 (0.67) |
| 321 | Eating disorder | 1 | 514 | 4197 | 4673759 | 2.17 (0.30, 15.41) | 2.16 (0.63) |
| 322 | Skin ulcer | 1 | 514 | 4216 | 4673740 | 2.16 (0.30, 15.34) | 2.15 (0.62) |
| 323 | Clostridium difficile infection | 1 | 514 | 4239 | 4673717 | 2.15 (0.30, 15.26) | 2.14 (0.61) |
| 324 | Conjunctivitis | 1 | 514 | 4296 | 4673660 | 2.12 (0.30, 15.06) | 2.11 (0.59) |
| 325 | Disturbance in attention | 2 | 513 | 8623 | 4669333 | 2.11 (0.53, 8.47) | 2.11 (1.16) |
| 326 | Ascites | 1 | 514 | 4413 | 4673543 | 2.06 (0.29, 14.66) | 2.06 (0.54) |
| 327 | Staphylococcal infection | 1 | 514 | 4492 | 4673464 | 2.02 (0.28, 14.40) | 2.02 (0.52) |
| 328 | Neuralgia | 1 | 514 | 4553 | 4673403 | 2.00 (0.28, 14.21) | 2.00 (0.50) |
| 329 | Nephrolithiasis | 2 | 513 | 9128 | 4668828 | 1.99 (0.50, 8.00) | 1.99 (0.99) |
| 330 | Crying | 1 | 514 | 4560 | 4673396 | 1.99 (0.28, 14.19) | 1.99 (0.49) |
| 331 | Gastrointestinal disorder | 4 | 511 | 18424 | 4659532 | 1.98 (0.74, 5.30) | 1.97 (1.92) |
| 332 | Feeding disorder | 1 | 514 | 4623 | 4673333 | 1.97 (0.28, 13.99) | 1.96 (0.47) |
| 333 | Crohn's disease | 3 | 512 | 14184 | 4663772 | 1.93 (0.62, 5.99) | 1.92 (1.33) |
| 334 | Neutrophil count decreased | 2 | 513 | 9499 | 4668457 | 1.92 (0.48, 7.68) | 1.91 (0.87) |
| 335 | Aphasia | 1 | 514 | 4747 | 4673209 | 1.92 (0.27, 13.63) | 1.91 (0.44) |
| 336 | Pruritus | 15 | 500 | 72748 | 4605208 | 1.90 (1.14, 3.17) | 1.87 (6.20) |
| 337 | Decreased appetite | 9 | 506 | 43577 | 4634379 | 1.89 (0.98, 3.66) | 1.88 (3.72) |
| 338 | Hepatic cytolysis | 1 | 514 | 4843 | 4673113 | 1.88 (0.26, 13.36) | 1.88 (0.41) |
| 339 | Gynaecomastia | 1 | 514 | 4915 | 4673041 | 1.85 (0.26, 13.16) | 1.85 (0.39) |
| 340 | Weight increased | 8 | 507 | 39761 | 4638195 | 1.84 (0.92, 3.70) | 1.83 (3.02) |
| 341 | Hypersomnia | 1 | 514 | 5091 | 4672865 | 1.79 (0.25, 12.70) | 1.78 (0.34) |
| 342 | Hepatotoxicity | 1 | 514 | 5192 | 4672764 | 1.75 (0.25, 12.46) | 1.75 (0.32) |
| 343 | Asthenia | 12 | 503 | 63218 | 4614738 | 1.74 (0.98, 3.09) | 1.72 (3.70) |
| 344 | Fatigue | 29 | 486 | 155427 | 4522529 | 1.74 (1.19, 2.53) | 1.69 (8.54) |
| 345 | Palmar-plantar erythrodysaesthesia syndrome | 1 | 514 | 5246 | 4672710 | 1.73 (0.24, 12.33) | 1.73 (0.31) |
| 346 | Pneumonitis | 1 | 514 | 5398 | 4672558 | 1.68 (0.24, 11.98) | 1.68 (0.28) |
| 347 | Cerebral haemorrhage | 1 | 514 | 5419 | 4672537 | 1.68 (0.24, 11.93) | 1.68 (0.27) |
| 348 | Thrombosis | 3 | 512 | 16733 | 4661223 | 1.63 (0.52, 5.08) | 1.63 (0.73) |
| 349 | Nausea | 24 | 491 | 136453 | 4541503 | 1.63 (1.08, 2.45) | 1.60 (5.53) |
| 350 | Movement disorder | 1 | 514 | 5647 | 4672309 | 1.61 (0.23, 11.45) | 1.61 (0.23) |
| 351 | Abdominal discomfort | 6 | 509 | 34222 | 4643734 | 1.60 (0.72, 3.58) | 1.59 (1.33) |
| 352 | Oropharyngeal pain | 3 | 512 | 17242 | 4660714 | 1.58 (0.51, 4.93) | 1.58 (0.64) |
| 353 | Constipation | 7 | 508 | 40746 | 4637210 | 1.57 (0.74, 3.31) | 1.56 (1.42) |
| 354 | Developmental delay | 1 | 514 | 5869 | 4672087 | 1.55 (0.22, 11.02) | 1.55 (0.19) |
| 355 | Limb injury | 1 | 514 | 6007 | 4671949 | 1.51 (0.21, 10.76) | 1.51 (0.17) |
| 356 | Laboratory test abnormal | 1 | 514 | 6064 | 4671892 | 1.50 (0.21, 10.66) | 1.50 (0.17) |
| 357 | Pneumonia | 9 | 506 | 55374 | 4622582 | 1.48 (0.77, 2.87) | 1.48 (1.40) |
| 358 | Rectal haemorrhage | 1 | 514 | 6131 | 4671825 | 1.48 (0.21, 10.55) | 1.48 (0.16) |
| 359 | Gastrooesophageal reflux disease | 2 | 513 | 12661 | 4665295 | 1.44 (0.36, 5.76) | 1.43 (0.26) |
| 360 | Vomiting | 12 | 503 | 76615 | 4601341 | 1.43 (0.81, 2.54) | 1.42 (1.53) |
| 361 | Intestinal obstruction | 1 | 514 | 6611 | 4671345 | 1.37 (0.19, 9.78) | 1.37 (0.10) |
| 362 | C-reactive protein increased | 1 | 514 | 6617 | 4671339 | 1.37 (0.19, 9.77) | 1.37 (0.10) |
| 363 | Eye disorder | 1 | 514 | 6681 | 4671275 | 1.36 (0.19, 9.68) | 1.36 (0.10) |
| 364 | Tinnitus | 1 | 514 | 6845 | 4671111 | 1.33 (0.19, 9.44) | 1.33 (0.08) |
| 365 | Dyspnoea exertional | 1 | 514 | 6900 | 4671056 | 1.32 (0.19, 9.37) | 1.32 (0.08) |
| 366 | Peripheral swelling | 5 | 510 | 34573 | 4643383 | 1.32 (0.55, 3.18) | 1.31 (0.38) |
| 367 | Pyrexia | 8 | 507 | 56130 | 4621826 | 1.30 (0.65, 2.61) | 1.29 (0.54) |
| 368 | Hypoglycaemia | 1 | 514 | 7032 | 4670924 | 1.29 (0.18, 9.19) | 1.29 (0.07) |
| 369 | Limb discomfort | 1 | 514 | 7058 | 4670898 | 1.29 (0.18, 9.16) | 1.29 (0.06) |
| 370 | Upper respiratory tract infection | 1 | 514 | 7141 | 4670815 | 1.27 (0.18, 9.05) | 1.27 (0.06) |
| 371 | Tachycardia | 2 | 513 | 14401 | 4663555 | 1.26 (0.31, 5.06) | 1.26 (0.11) |
| 372 | Rash erythematous | 1 | 514 | 7331 | 4670625 | 1.24 (0.17, 8.82) | 1.24 (0.05) |
| 373 | Drug-induced liver injury | 1 | 514 | 7392 | 4670564 | 1.23 (0.17, 8.74) | 1.23 (0.04) |
| 374 | Heart rate decreased | 1 | 514 | 7483 | 4670473 | 1.21 (0.17, 8.64) | 1.21 (0.04) |
| 375 | Nervousness | 1 | 514 | 7517 | 4670439 | 1.21 (0.17, 8.60) | 1.21 (0.04) |
| 376 | Pancreatitis | 1 | 514 | 7552 | 4670404 | 1.20 (0.17, 8.56) | 1.20 (0.03) |
| 377 | Hepatic function abnormal | 1 | 514 | 7572 | 4670384 | 1.20 (0.17, 8.54) | 1.20 (0.03) |
| 378 | Headache | 14 | 501 | 107480 | 4570476 | 1.19 (0.70, 2.02) | 1.18 (0.41) |
| 379 | Platelet count decreased | 3 | 512 | 23159 | 4654797 | 1.18 (0.38, 3.66) | 1.18 (0.08) |
| 380 | Back pain | 5 | 510 | 38876 | 4639080 | 1.17 (0.48, 2.82) | 1.17 (0.12) |
| 381 | Pain in extremity | 6 | 509 | 47920 | 4630036 | 1.14 (0.51, 2.55) | 1.14 (0.10) |
| 382 | Dyspepsia | 2 | 513 | 16053 | 4661903 | 1.13 (0.28, 4.54) | 1.13 (0.03) |
| 383 | Alanine aminotransferase increased | 1 | 514 | 8089 | 4669867 | 1.12 (0.16, 7.99) | 1.12 (0.01) |
| 384 | Cellulitis | 1 | 514 | 8113 | 4669843 | 1.12 (0.16, 7.97) | 1.12 (0.01) |
| 385 | Lethargy | 1 | 514 | 8345 | 4669611 | 1.09 (0.15, 7.74) | 1.09 (0.01) |
| 386 | Heart rate increased | 2 | 513 | 16704 | 4661252 | 1.09 (0.27, 4.36) | 1.09 (0.01) |
| 387 | Death | 26 | 489 | 218109 | 4459847 | 1.09 (0.73, 1.61) | 1.08 (0.17) |
| 388 | Fluid retention | 1 | 514 | 8494 | 4669462 | 1.07 (0.15, 7.61) | 1.07 (0.00) |
| 389 | Leukopenia | 1 | 514 | 8610 | 4669346 | 1.06 (0.15, 7.51) | 1.05 (0.00) |
| 390 | Cognitive disorder | 1 | 514 | 8798 | 4669158 | 1.03 (0.15, 7.34) | 1.03 (0.00) |
| 391 | Abdominal pain upper | 4 | 511 | 35560 | 4642396 | 1.02 (0.38, 2.73) | 1.02 (0.00) |
| 392 | Swelling | 2 | 513 | 17806 | 4660150 | 1.02 (0.25, 4.09) | 1.02 (0.00) |
| 393 | Flatulence | 1 | 514 | 9189 | 4668767 | 0.99 (0.14, 7.03) | 0.99 (0.00) |
| 394 | Bradycardia | 1 | 514 | 9340 | 4668616 | 0.97 (0.14, 6.92) | 0.97 (0.00) |
| 395 | Erythema | 3 | 512 | 28767 | 4649189 | 0.95 (0.30, 2.95) | 0.95 (0.01) |
| 396 | Seizure | 3 | 512 | 28824 | 4649132 | 0.95 (0.30, 2.94) | 0.95 (0.01) |
| 397 | Gait inability | 1 | 514 | 9770 | 4668186 | 0.93 (0.13, 6.61) | 0.93 (0.01) |
| 398 | Blood pressure increased | 3 | 512 | 29465 | 4648491 | 0.92 (0.30, 2.88) | 0.92 (0.02) |
| 399 | Renal disorder | 1 | 514 | 9828 | 4668128 | 0.92 (0.13, 6.57) | 0.92 (0.01) |
| 400 | Influenza | 2 | 513 | 19901 | 4658055 | 0.91 (0.23, 3.66) | 0.91 (0.02) |
| 401 | Bronchitis | 1 | 514 | 10315 | 4667641 | 0.88 (0.12, 6.26) | 0.88 (0.02) |
| 402 | Insomnia | 4 | 511 | 41821 | 4636135 | 0.87 (0.32, 2.32) | 0.87 (0.08) |
| 403 | Influenza like illness | 1 | 514 | 10621 | 4667335 | 0.85 (0.12, 6.08) | 0.86 (0.02) |
| 404 | Inflammation | 1 | 514 | 10704 | 4667252 | 0.85 (0.12, 6.03) | 0.85 (0.03) |
| 405 | Blood creatinine increased | 1 | 514 | 10882 | 4667074 | 0.83 (0.12, 5.94) | 0.83 (0.03) |
| 406 | Blood pressure decreased | 1 | 514 | 11218 | 4666738 | 0.81 (0.11, 5.76) | 0.81 (0.04) |
| 407 | Nasopharyngitis | 3 | 512 | 33681 | 4644275 | 0.81 (0.26, 2.51) | 0.81 (0.14) |
| 408 | Cough | 5 | 510 | 56101 | 4621855 | 0.81 (0.33, 1.95) | 0.81 (0.23) |
| 409 | Injury | 1 | 514 | 11241 | 4666715 | 0.81 (0.11, 5.75) | 0.81 (0.05) |
| 410 | Burning sensation | 1 | 514 | 11316 | 4666640 | 0.80 (0.11, 5.71) | 0.80 (0.05) |
| 411 | Nasal congestion | 1 | 514 | 11349 | 4666607 | 0.80 (0.11, 5.69) | 0.80 (0.05) |
| 412 | Dry eye | 1 | 514 | 11395 | 4666561 | 0.80 (0.11, 5.67) | 0.80 (0.05) |
| 413 | Cardiac arrest | 1 | 514 | 11851 | 4666105 | 0.77 (0.11, 5.45) | 0.77 (0.07) |
| 414 | Hypoaesthesia | 2 | 513 | 23753 | 4654203 | 0.76 (0.19, 3.06) | 0.76 (0.15) |
| 415 | Depression | 3 | 512 | 36116 | 4641840 | 0.75 (0.24, 2.34) | 0.75 (0.24) |
| 416 | Wheezing | 1 | 514 | 12061 | 4665895 | 0.75 (0.11, 5.35) | 0.75 (0.08) |
| 417 | Haematochezia | 1 | 514 | 12069 | 4665887 | 0.75 (0.11, 5.35) | 0.75 (0.08) |
| 418 | Illness | 4 | 511 | 48838 | 4629118 | 0.74 (0.28, 1.98) | 0.74 (0.36) |
| 419 | Hepatic enzyme increased | 1 | 514 | 12263 | 4665693 | 0.74 (0.10, 5.27) | 0.74 (0.09) |
| 420 | White blood cell count decreased | 2 | 513 | 25214 | 4652742 | 0.72 (0.18, 2.88) | 0.72 (0.22) |
| 421 | Rhinorrhoea | 1 | 514 | 12688 | 4665268 | 0.72 (0.10, 5.09) | 0.72 (0.11) |
| 422 | Flushing | 1 | 514 | 12738 | 4665218 | 0.71 (0.10, 5.07) | 0.71 (0.12) |
| 423 | Cerebrovascular accident | 2 | 513 | 26371 | 4651585 | 0.69 (0.17, 2.76) | 0.69 (0.28) |
| 424 | Hallucination | 1 | 514 | 13333 | 4664623 | 0.68 (0.10, 4.84) | 0.68 (0.15) |
| 425 | Paraesthesia | 2 | 513 | 26813 | 4651143 | 0.68 (0.17, 2.71) | 0.68 (0.31) |
| 426 | Urticaria | 2 | 513 | 27226 | 4650730 | 0.67 (0.17, 2.67) | 0.67 (0.33) |
| 427 | Visual impairment | 2 | 513 | 28006 | 4649950 | 0.65 (0.16, 2.60) | 0.65 (0.38) |
| 428 | Infection | 2 | 513 | 28484 | 4649472 | 0.64 (0.16, 2.55) | 0.64 (0.41) |
| 429 | Malaise | 5 | 510 | 71042 | 4606914 | 0.64 (0.26, 1.53) | 0.64 (1.03) |
| 430 | Sleep disorder | 1 | 514 | 14942 | 4663014 | 0.61 (0.09, 4.32) | 0.61 (0.25) |
| 431 | Cardiac failure | 1 | 514 | 15266 | 4662690 | 0.59 (0.08, 4.23) | 0.60 (0.28) |
| 432 | Syncope | 1 | 514 | 15765 | 4662191 | 0.58 (0.08, 4.09) | 0.58 (0.31) |
| 433 | Gait disturbance | 2 | 513 | 32928 | 4645028 | 0.55 (0.14, 2.21) | 0.55 (0.73) |
| 434 | Abdominal distension | 1 | 514 | 16818 | 4661138 | 0.54 (0.08, 3.84) | 0.54 (0.39) |
| 435 | Haemoglobin decreased | 1 | 514 | 17046 | 4660910 | 0.53 (0.07, 3.78) | 0.53 (0.41) |
| 436 | Musculoskeletal stiffness | 1 | 514 | 17120 | 4660836 | 0.53 (0.07, 3.77) | 0.53 (0.42) |
| 437 | Weight decreased | 3 | 512 | 51789 | 4626167 | 0.52 (0.17, 1.63) | 0.53 (1.29) |
| 438 | Pain | 10 | 505 | 170615 | 4507341 | 0.52 (0.28, 0.98) | 0.53 (4.26) |
| 439 | Somnolence | 2 | 513 | 35693 | 4642263 | 0.51 (0.13, 2.03) | 0.51 (0.95) |
| 440 | Sinusitis | 1 | 514 | 17993 | 4659963 | 0.50 (0.07, 3.58) | 0.50 (0.49) |
| 441 | Migraine | 1 | 514 | 18543 | 4659413 | 0.49 (0.07, 3.48) | 0.49 (0.53) |
| 442 | Palpitations | 1 | 514 | 18838 | 4659118 | 0.48 (0.07, 3.42) | 0.48 (0.56) |
| 443 | Renal impairment | 1 | 514 | 18998 | 4658958 | 0.48 (0.07, 3.39) | 0.48 (0.57) |
| 444 | Hypertension | 2 | 513 | 38033 | 4639923 | 0.48 (0.12, 1.91) | 0.48 (1.15) |
| 445 | Dehydration | 1 | 514 | 19093 | 4658863 | 0.47 (0.07, 3.38) | 0.48 (0.58) |
| 446 | Loss of consciousness | 1 | 514 | 19470 | 4658486 | 0.47 (0.07, 3.31) | 0.47 (0.61) |
| 447 | Cardiac disorder | 1 | 514 | 19611 | 4658345 | 0.46 (0.06, 3.29) | 0.46 (0.62) |
| 448 | Thrombocytopenia | 1 | 514 | 19711 | 4658245 | 0.46 (0.06, 3.27) | 0.46 (0.63) |
| 449 | Chills | 1 | 514 | 19910 | 4658046 | 0.46 (0.06, 3.24) | 0.46 (0.65) |
| 450 | Neuropathy peripheral | 1 | 514 | 20672 | 4657284 | 0.44 (0.06, 3.12) | 0.44 (0.72) |
| 451 | Feeling abnormal | 2 | 513 | 42787 | 4635169 | 0.42 (0.11, 1.69) | 0.42 (1.57) |
| 452 | Chronic kidney disease | 1 | 514 | 23225 | 4654731 | 0.39 (0.05, 2.77) | 0.39 (0.95) |
| 453 | Renal failure | 1 | 514 | 24714 | 4653242 | 0.37 (0.05, 2.61) | 0.37 (1.09) |
| 454 | Chest pain | 1 | 514 | 24997 | 4652959 | 0.36 (0.05, 2.58) | 0.36 (1.12) |
| 455 | Tremor | 1 | 514 | 25214 | 4652742 | 0.36 (0.05, 2.55) | 0.36 (1.14) |
| 456 | Dizziness | 3 | 512 | 83749 | 4594207 | 0.32 (0.10, 1.00) | 0.33 (4.27) |
| 457 | Memory impairment | 1 | 514 | 28247 | 4649709 | 0.32 (0.05, 2.28) | 0.32 (1.44) |
| 458 | Anxiety | 2 | 513 | 56539 | 4621417 | 0.32 (0.08, 1.28) | 0.32 (2.90) |
| 459 | Fall | 2 | 513 | 57079 | 4620877 | 0.32 (0.08, 1.27) | 0.32 (2.96) |
| 460 | Muscle spasms | 1 | 514 | 28686 | 4649270 | 0.32 (0.04, 2.24) | 0.32 (1.48) |
| 461 | Blood glucose increased | 1 | 514 | 31749 | 4646207 | 0.28 (0.04, 2.03) | 0.29 (1.79) |
| 462 | Hypersensitivity | 1 | 514 | 33257 | 4644699 | 0.27 (0.04, 1.93) | 0.27 (1.95) |
| 463 | Hypotension | 1 | 514 | 35559 | 4642397 | 0.25 (0.04, 1.81) | 0.26 (2.19) |
| 464 | Drug hypersensitivity | 1 | 514 | 36187 | 4641769 | 0.25 (0.04, 1.78) | 0.25 (2.25) |
| 465 | Acute kidney injury | 1 | 514 | 36552 | 4641404 | 0.25 (0.03, 1.76) | 0.25 (2.29) |
| 466 | Arthralgia | 2 | 513 | 84307 | 4593649 | 0.21 (0.05, 0.85) | 0.22 (5.82) |
| 467 | Dyspnoea | 2 | 513 | 97307 | 4580649 | 0.18 (0.05, 0.74) | 0.19 (7.24) |

Note: The first 53 items are positive signals.

Table S2: Selumetinib-related reports extracted from FAERS that were not included in the analyses of this study

| No. | PT | *a* | *b* | *c* | *d* |
| --- | --- | --- | --- | --- | --- |
| 1 | Tumour pain | 5 | 510 | 261 | 4677695 |
| 2 | Neoplasm recurrence | 3 | 512 | 720 | 4677236 |
| 3 | Neoplasm | 11 | 504 | 2768 | 4675188 |
| 4 | Neoplasm progression | 11 | 504 | 12392 | 4665564 |
| 5 | Malignant neoplasm progression | 10 | 505 | 24168 | 4653788 |
| 6 | Tumour inflammation | 1 | 514 | 24 | 4677932 |
| 7 | Neoplasm malignant | 5 | 510 | 24770 | 4653186 |
| 8 | Neurofibromatosis | 5 | 510 | 31 | 4677925 |
| 9 | Drug ineffective | 12 | 503 | 293557 | 4384399 |
| 10 | Disease progression | 11 | 504 | 29308 | 4648648 |
| 11 | Condition aggravated | 2 | 513 | 69983 | 4607973 |
| 12 | Adverse drug reaction | 1 | 514 | 23446 | 4654510 |
| 13 | Drug ineffective for unapproved indication | 1 | 514 | 15116 | 4662840 |
| 14 | Drug intolerance | 1 | 514 | 23205 | 4654751 |
| 15 | No adverse event | 1 | 514 | 46509 | 4631447 |
| 16 | Symptom recurrence | 1 | 514 | 2077 | 4675879 |
| 17 | Therapeutic product effect decreased | 1 | 514 | 15551 | 4662405 |
| 18 | Therapy partial responder | 1 | 514 | 5361 | 4672595 |
| 19 | Treatment failure | 1 | 514 | 31763 | 4646193 |
| 20 | Treatment noncompliance | 1 | 514 | 11242 | 4666714 |
| 21 | Vascular complication associated with device | 1 | 514 | 2 | 4677954 |
| 22 | COVID-19 | 4 | 511 | 95278 | 4582678 |
| 23 | Off label use | 43 | 472 | 241831 | 4436125 |
| 24 | Product dose omission issue | 10 | 505 | 135654 | 4542302 |
| 25 | Toxicity to various agents | 9 | 506 | 36330 | 4641626 |
| 26 | Product use in unapproved indication | 4 | 511 | 79760 | 4598196 |
| 27 | Accidental overdose | 1 | 514 | 6695 | 4671261 |
| 28 | Intentional dose omission | 1 | 514 | 8835 | 4669121 |
| 29 | Intentional overdose | 1 | 514 | 14074 | 4663882 |
| 30 | Intentional product misuse | 1 | 514 | 18463 | 4659493 |
| 31 | Overdose | 1 | 514 | 81629 | 4596327 |
| 32 | Product dose omission in error | 1 | 514 | 11354 | 4666602 |
| 33 | Product prescribing error | 1 | 514 | 9285 | 4668671 |
| 34 | Product prescribing issue | 1 | 514 | 6829 | 4671127 |
| 35 | Product use issue | 1 | 514 | 41653 | 4636303 |
| 36 | Underdose | 1 | 514 | 12202 | 4665754 |
| 37 | Wrong technique in product usage process | 1 | 514 | 66253 | 4611703 |
| 38 | Neurofibrosarcoma | 11 | 504 | 24 | 4677932 |
| 39 | Neurofibroma | 9 | 506 | 20 | 4677936 |
| 40 | Fibroma | 2 | 513 | 127 | 4677829 |
| 41 | Product availability issue | 1 | 514 | 10640 | 4667316 |
| 42 | Product distribution issue | 1 | 514 | 2802 | 4675154 |
| 43 | Intentional self-injury | 1 | 514 | 3558 | 4674398 |
| 44 | Insurance issue | 4 | 511 | 4187 | 4673769 |
| 45 | Therapy cessation | 2 | 513 | 10015 | 4667941 |
| 46 | Nail operation | 1 | 514 | 78 | 4677878 |
| 47 | Surgery | 1 | 514 | 12678 | 4665278 |
| 48 | Therapy interrupted | 1 | 514 | 31191 | 4646765 |
